# Investigating the relationship between affective valence and reinforcement learning

**DOI:** 10.1016/j.isci.2026.116863

**Published:** 2026-07-22

**Authors:** Daniel F. Parr, Seth Madlon-Kay, Gregory R. Samanez-Larkin, Kevin S. LaBar

**Affiliations:** 1 Department of Psychology & Neuroscience, Duke University, Durham, NC 27708, USA; 2 Department of Neurobiology, Duke University, Durham, NC 27708, USA

**Keywords:** emotion, valence, affect, reinforcement learning, decision making, computational modeling, prediction error, reward

## Abstract

Affective valence and reinforcement learning (RL) are increasingly recognized to be closely connected, yet the exact nature of their relationship remains unclear. Here, we investigated how RL-related computations contribute to affective valence, and how affective valence, in turn, contributes to RL. Applying an original computational method, we found that affective experience during RL tasks was best explained by a combination of three prominent theoretical perspectives: valence is determined by reward, prediction errors, and counterfactual comparisons. Further, we found that actions were reinforced by affective responses in addition to external rewards: participants preferred choice options that led to more positive affect, in addition to preferring options that led to greater reward. Altogether, our results illuminate the role of RL computations in affective experience and of affect in RL, providing mechanistic insight into affect, learning, and choice. Moreover, our studies validate a powerful new computational framework for future research on these topics.

## Highlights

• We develop an original data analysis method for testing overlapping hypotheses
• Multiple distinct reinforcement learning computations contribute to affective valence
• Behavior is reinforced by affective responses in addition to external rewards

## Graphical abstract

**Figure:**
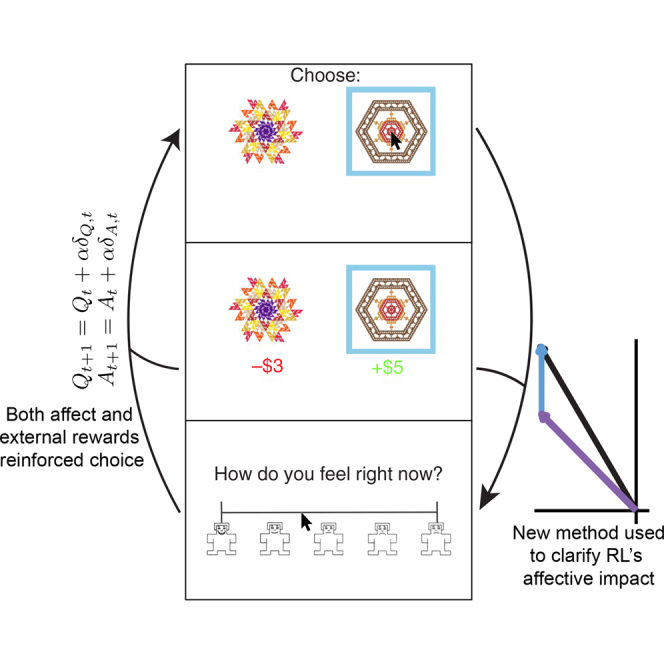

## Teaser

Cognitive neuroscience; Computer science; Psychology

## Introduction

Affective valence—the subjective pleasantness or unpleasantness of emotion—is a core feature of emotional experience. A related phenomenon is reinforcement learning (RL)—learning what to do to maximize reward (i.e., good outcomes)^1^—which is a central mechanism underlying value-based choice. It is increasingly recognized that affect and RL are closely connected, yet the exact nature of their relationship remains unclear. Here, we sought to illuminate this relationship, investigating the ways in which RL-related computations contribute to affective valence and in which affective valence contributes to RL. A greater understanding of this bidirectional relationship is relevant not only to affective and decision science but also to many other areas of human psychology: affect and RL play key roles in mental health,^2^^,^^3^^,^^4^ emotion regulation,^5^^,^^6^ social behavior,^7^^,^^8^^,^^9^^,^^10^ and a variety of cognitive processes.^11^^,^^12^^,^^13^^,^^14^

We began by considering how RL-related computations determine affective valence. Numerous hypotheses have been proposed on this topic, including hypotheses that explicitly predict effects of RL variables on valence as well as those that do not use RL terminology but which can readily be translated into this framework (e.g., that discuss “good outcomes” rather than “rewards”). Many of these hypotheses fall into one of three groups.

A first group of hypotheses claims that the more rewarding a stimulus is, or the greater reward it predicts, the better this stimulus makes us feel.^15^^,^^16^^,^^17^ For example, it is more pleasurable to eat food we crave than food we dislike, or to receive a large raise than to receive a small one. We will refer to this perspective as the *reward theory*.

A second group of hypotheses holds that the more rewarding a stimulus is relative to the expected reward, the better it makes us feel.^18^^,^^19^^,^^20^^,^^21^ We will refer to this perspective as the *prediction error theory*. For example, an employee who expects a promotion might be saddened to be passed over, while a co-worker who expects to be laid off might be elated to keep their current position. Expected reward values can also be adjusted in hindsight,^19^^,^^22^ such that an employee might feel less angst about being passed over if they decide, in retrospect, that a promotion was always a longshot.

A final set of hypotheses claims that the more rewarding a stimulus is relative to what could have been obtained with different choices, the better it makes us feel.^23^^,^^24^^,^^25^^,^^26^^,^^27^ For example, an investor may be pleased with a stock that yields modest returns during a market slump in which alternative stocks plummet, yet be frustrated by stocks that yield modest returns during a market surge. This will be referred to as the *counterfactual comparison theory*. Here, we use “counterfactual” to mean an outcome that could have been obtained with different actions, rather than any outcome that could conceivably have occurred (as the term is sometimes used).

Although these theories are qualitatively distinct, their predictions overlap. All theories predict that receiving greater reward will elicit more positive valence. Moreover, counterfactual outcomes can inform expectations (e.g., a market slump implies a high likelihood of losing money) and expectations can serve as counterfactuals (e.g., a passed-over employee may be regretful if they expect they would have been promoted had they worked harder), meaning that both prediction error and counterfactual comparison hypotheses can predict negative effects of expectations and counterfactuals on valence.^19^^,^^25^ As a result of these overlaps, evidence used to support one theory can typically be explained by others. To determine the true relationship between RL computations and valence, a method that can parse evidence for these confounded theories is needed.

Here, we offer an original computational framework for testing theories whose predictions overlap in this way. We used this framework to analyze data from RL tasks in which participants intermittently rated their affective valence, and in which counterfactual outcomes were shown (Figures 3 and 6). We used computational models to estimate the effects of several RL variables (e.g., reward, expected reward, counterfactual reward) on valence. The predictions of the three theories overlap, such that they often predict effects of the same RL variables; e.g., every theory predicts a positive effect of reward on valence. However, these theories predict distinct *patterns* of effects across the set of RL variables. For example, the prediction error theory, unlike the reward or counterfactual comparison theories, predicts that the effect of expected reward will be equal and opposite to the effect of the reward received.

**Figure 1:**
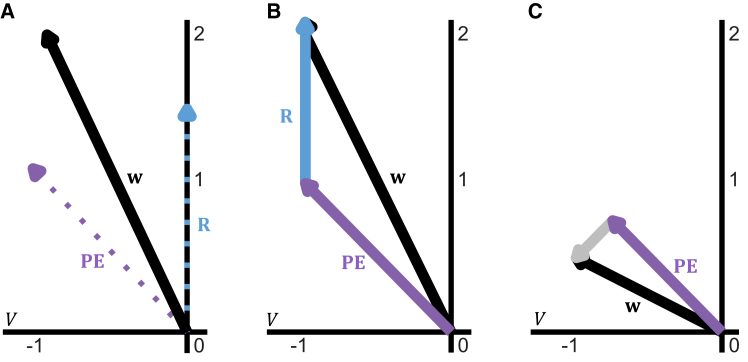
Example effect and direction vectors Consider an RL task in which, on each trial, participants receive an outcome of reward value *r*, having expected an outcome of reward value *V*. (A) The effects of reward *r* and expected reward *V* on affective valence can be estimated from the data and represented in a two-dimensional vector **w**. The reward theory predicts that **w** will align with the direction vector **R**; i.e., it predicts a positive effect of *r*. The prediction error theory predicts that **w** will align with the direction vector **PE**; i.e., it predicts a positive effect of *r* and a negative effect of *V* that is of equal magnitude. (B) To identify the theory or combination of theories that best accounts for the effects, lengths are assigned to **R** and **PE** so that their sum approximates **w** as closely as possible. Here, the effects encoded in **w** are best explained by a combination of both theories’ predictions. (C) A different scenario in which the effects are best explained by prediction error alone: **w** is best approximated by assigning positive length to **PE** only. The gray vector represents the residual from this approximation.

The distinct patterns predicted by each theory can be represented as *direction vectors* (i.e., vectors that point in a fixed direction but do not have a fixed length) in a space defined by RL variables (Figure 1A). The actual effects of these RL variables on valence can be represented as a vector in the same space. To identify the theory—or weighted combination of theories—that best explains how RL-related computations contribute to valence, lengths can be assigned to the direction vectors (i.e., weights can be assigned to each theory) such that their sum approximates the vector of RL variable effects as closely as possible (Figures 1B and 1C). We used this method in the present studies, finding that the RL variable effects were best described by a combination of all three theories: reward, prediction errors, and counterfactual comparisons each contributed to affective valence.

Measuring and modeling affective experience during RL not only allowed us to investigate the determinants of valence but also provided an opportunity to examine affective influences on learning and choice. Therefore, after assessing the contributions of RL computations to affect, we next investigated the contributions of affect to RL.

Standard RL models assume that behavior is reinforced by external rewards (e.g., money and food), such that actions which lead to greater reward are more likely to be repeated. Here, we define a *reward*—or, interchangeably, an *external reward*—as a desirable external stimulus, such as money or palatable food. By definition, stimuli with greater reward value are preferred to those with less (thus, stimuli with negative reward value are undesirable and avoided). This definition closely matches the common usage of the term *reward* in the human RL literature (where reward is typically operationalized as money). Although *reward* is sometimes defined more broadly as whatever choices optimize, we adopt a more specific conceptualization of rewards as external reinforcers here.

Although standard models recognize the influence of external reward on behavior, they do not explicitly identify a role for affect. Here, we hypothesized that affect reinforces choice alongside external reward, such that a person is more likely to repeat actions that have led to more positive affect, in addition to preferring actions that have led to greater reward. This hypothesis was motivated by past theories which argue that decision options (i.e., actions) associated with more affectively pleasant outcomes are more likely to be selected—e.g., because decision-makers seek to maximize the pleasantness of their affect,^28^^,^^29^^,^^30^ or because affect signals the values of outcomes and actions.^31^^,^^32^ In the context of RL, where action-outcome associations are acquired through experience, this view suggests that actions which typically lead to more positive affect are more likely to be repeated; i.e., that affect reinforces behavior. Thus, whether an action is repeated may depend not merely on how much reward it yields but also on affective responses to those rewards. For example, whether an investor repeats a financial decision (e.g., to buy stock from a particular company) might depend not only on how large the previous returns from that investment were but also on how positively or negatively those returns made them feel.

Conceivably, actions could be reinforced by affect alone, without any additional effect of external reward. However, given that extensive research on human RL demonstrates a strong connection between the rewards an action has typically yielded and its likelihood of being repeated, we considered it likely that reward *per se* shapes behavior even after accounting for the influence of affect (e.g., that the choice to reinvest in a company depends on the size of previous returns in addition to how those returns made the investor feel). Indeed, consistent with this possibility, recent studies of social decision-making show that affective valence and monetary reward can have independent effects on choice.^7^^,^^33^ Therefore, we hypothesized that actions are reinforced by both affect and external reward.

Although past literature supports the plausibility of our hypothesis, it has not previously been tested. Therefore, we examined the effects of affect and monetary reward on learning and choice during three RL tasks. We found consistent evidence that participants preferred choice options which led to more positive affect in addition to preferring options that led to greater reward—confirming our hypothesis that affect reinforces choice alongside reward *per se*. Thus, our studies not only illuminate the role of RL computations in affect, but also identify a key role for affect in RL.

## Results

### Study 1: Exploring the determinants of affective valence

**Figure 2:**
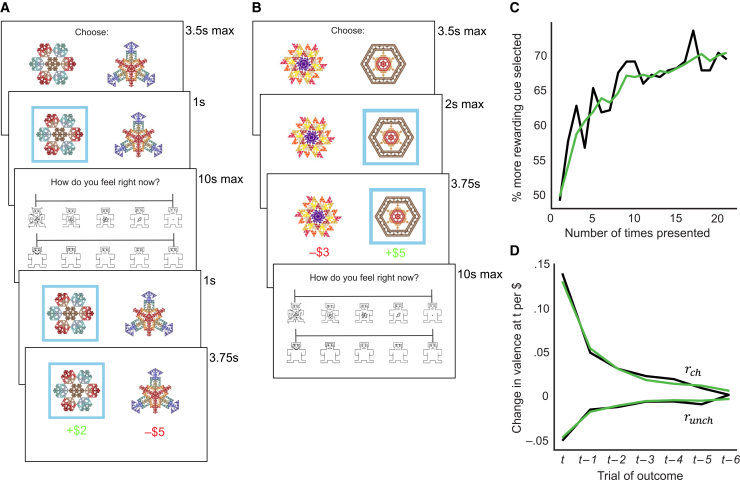
Study 1 task and model Participants completed a two-armed bandit task with affect probes. On each trial, they were presented with a pair of cues, chose between them, and then viewed the outcome of each cue (a monetary win or loss ranging from -$5 to +$5). Six pairs of cues were presented 21 times each. (A) On one-third of trials, participants rated the valence and arousal of their current affective state after choosing a cue. We only consider valence data in this article. (B) On a separate third of trials, participants completed an affect rating after viewing the cue outcomes. On the remaining third of trials, no affect ratings were completed. The three trial types were pseudorandomly interleaved. (C) Observed and model-predicted learning curves. The black line shows the percentage of trials on which participants chose the more rewarding cue (i.e., the cue that yielded greater reward on average), given the number of times the cue pair had been presented. The green line shows the *Q*-learning model’s prediction of these percentages (predictions were conditioned on participants’ actual past choices and rewards, not on model-simulated choice and reward histories). The close correspondence between these curves suggests that the model accurately describes learning in this task. (D) Effect of outcomes on valence ratings at trial *t*. The black lines show coefficients from a regression of valence ratings onto rewards from the chosen cue (top line, *r*_*ch*_) and unchosen cue (bottom line, *r*_*unch*_) at different time points. The green lines show coefficients from the same regression fit to model-predicted ratings. Thus, the model accurately describes effects of outcomes on affective valence over time.

Participants (*N* = 101) completed a two-armed bandit task (Figure 2). On each trial, they chose between two cues (fractal images) and then viewed the outcomes of both cues. Each cue was probabilistically associated with a range of monetary outcomes; these probabilities stayed constant across trials. In addition to making choices between cues, participants rated the valence of their current affect regularly throughout the task.

We constructed a hierarchical Bayesian computational model to estimate the effects of several RL variables on valence ratings: the monetary reward from the chosen cue *r*_*ch*_ and unchosen cue *r*_*unch*_, the expected reward from the chosen cue *Q*_*ch*_ and unchosen cue *Q*_*unch*_, the expected reward from a trial in the current block *V*_*block*_, and the expected value of the current trial (given the cue outcomes) *V*_*trial*_. To estimate expectations, we modeled participants’ choices using a *Q*-learning algorithm—the standard RL algorithm for modeling human bandit task data.^34^ This model assumes that participants estimate the expected value *Q* (i.e., the reward association) of each cue *c*, updating this estimate on every trial in which an outcome from the cue is shown:

(Equation 1) $$Q_{c,t}=Q_{c,t-1}+\alpha \left(r_{c,t-1}-Q_{c,t-1}\right)$$

where *t* is the trial number and *α* is the learning rate (a free parameter constrained to between 0 and 1). These *Q* values were then used to predict choice such that the probability of choosing cue *a* over cue *b* on trial *t* was modeled as:

(Equation 2) $$p{\left(\text{choose}\;a\right)}_{t}=\sigma \left(\beta_{Q}\left(Q_{a,t}-Q_{b,t}\right)+\beta_{S}\left(S_{a,t}-S_{b,t}\right)\right)$$

where *S* is a nuisance variable capturing autocorrelation in choices, *σ* is the logistic function (used to transform values to the 0–1 probability scale), and *β*_*Q*_ and *β*_*S*_ are the effects of *Q* and *S* on choice, respectively. Although we later consider affective influences on choice, we did not estimate these influences here since our goal in fitting this RL model was simply to estimate reward expectations. This model was chosen over alternatives via a model selection procedure that is described in the supplemental information (Data S1). The learning curve predicted by this model closely matched our data (Figure 2C), indicating that it provided a good description of learning in this task.

By fitting this model to our data, we acquired estimates of *Q*_*ch*_ and *Q*_*unch*_ on each trial and could also estimate *V*_*block*_ and *V*_*trial*_ as follows:

(Equation 3) $$V_{block,t}=V_{block,t-1}+\alpha \left(r_{ch,t-1}-V_{block,t-1}\right)$$

(Equation 4) $$V_{trial,t}=r_{ch,t}\cdot p{\left(\text{choice}\right)}_{t}+r_{unch,t}\left(1-p{\left(\text{choice}\right)}_{t}\right)$$

where *p*(choice)_*t*_ is the model-estimated probability of the choice made on *t*. Note that *V*_*trial*_ (which represents the expected value of the trial, given the outcomes) was proposed to influence valence by Bennett and colleagues^19^ (also see Emanuel and Eldar^35^ for a closely related proposal); specifically, they proposed that affective responses to outcomes are influenced by *r*_*ch*_-*V*_*trial*_ (“policy-weighted reward differences,” Table 1). Although it may seem counter-intuitive that choice probabilities influence valence after the choice and outcome are known, Bennett et al. provide theoretical and empirical reasons to expect that this quantity shapes the affective impact of outcomes (e.g., the valence elicited by an outcome often depends on the probability of the choice that led to it, as do RL quantities responsible for policy updates). We also note that, although our task doesn’t explicitly require participants to learn *V*_*block*_, past RL research shows that participants learn how much reward to expect from a given state (e.g., from a trial in a given block) even when not required to do so.^36^^,^^37^^,^^38^^,^^39^ Additionally, we show in the supplemental information that replacing *V*_*block*_ with different estimates of reward expectations does not change our results (Data S3).

**Table 1:** Valence predictions

	Reward theory	Prediction error theory	Counterfactual comparison theory
Choice	*R_choice_* = *Q_ch_*	*PE*_*choice*_ = *Q*_*ch*_-*V*_*block*_	*CC*_*choice*_ = *Q*_*ch*_-*Q*_*unch*_
Outcome	*R*_*out*_ = *r*_*ch*_	*PE*_*out*,*Q*_ = *r*_*ch*_-*Q*_*ch*_ *PE*_*out*,*V*_ = *r*_*ch*_-*V*_*trial*_	*CC*_*out*_ = *r*_*ch*_-*r*_*unch*_

Variables predicted to have positive effects on the valence elicited by choices (top row) and outcomes (bottom row) by hypotheses associated with each theory. We refer to the terms on the right-hand sides of these equations as “RL variables.” *Q*_*ch*_: the expected monetary reward from the chosen cue, *r*_*ch*_: the reward from the chosen cue, *V*_*block*_: the reward generally expected on a trial in the current block (therefore, *PE*_*choice*_ represents a temporal difference error), and *V*_*trial*_: the expected value of the trial, given the outcomes that were received (predicted to influence valence by Bennett and colleagues^19^), *Q*_*unch*_: the expected reward from the unchosen cue, *r*_*unch*_: the reward from the unchosen cue. Note that the prediction error *r*_*ch*_-*V*_*block*_, shown in Equation 3, does not appear in this table because it is already implied by *PE*_*ch*__*o_ice_*_ and *PE*_*out*,*Q*_, which sum to *r*_*ch*_-*V*_*block*_.

To predict valence ratings, we assumed that affect was impacted by two events on each trial: the choice of a cue and the receipt of an outcome. Although it is conceivable that the presentation of cues also impacted affect, we show in the supplemental information that the data are best modeled without this effect, and that its inclusion would not change our results (Data S4). Each theory makes different predictions about how choices and outcomes will impact valence, which are displayed in Table 1. Following the predictions of the three theories, we assumed that *V*_*block*_, *Q*_*ch*_, and *Q*_*unch*_ would influence the affective response to the chosen cue, and that *r*_*ch*_, *r*_*unch*_, *Q*_*ch*_, and *V*_*trial*_ would influence the affective response to the outcome. Consistent with several previous studies,^40^^,^^41^^,^^42^^,^^43^ we assumed that the affective impacts of choices and outcomes decayed exponentially across trials, such that the valence rating *valence* on trial *t* was given by:

(Equation 5) $$valence_{t}=w_{x}\cdot \sum_{i=1}^{t}\gamma^{t-i}x_{i}+w_{z}\cdot z_{t}+\epsilon_{t}\;\;x_{i}={\left[V_{block,i},Q_{ch,choice,i},Q_{unch,i},V_{trial,i},Q_{ch,out,i},r_{ch,i},r_{unch,i}\right]}^{⊤}$$

where *γ* is the decay rate (constrained to between 0 and 1), **z** is a vector of nuisance predictors, and **w**_**x**_ and **w**_**z**_ contain the effects of **x** and **z**, respectively (see STAR Methods for further details). The assumption of exponentially decaying effects closely matched our data (Figure 2D), and provided a better model fit than alternative assumptions (see Data S2 for model comparisons). Overall, the model fit valence ratings well: *R*^2^ = 0.29 for ratings made after choices, *R*^2^ = 0.44 for ratings made after outcomes (*R*^2^ > 0.26 is considered a large amount of variance explained^44^). The estimated effects of RL variables on valence are shown in Figure S8.

To test the three theories, we represented each theory’s predictions as direction vectors (Table 2). To approximate the effects of RL variables on valence **w**_**x**_, we assigned lengths (Manhattan norms) **m** to each direction vector and calculated their sum ${\hat{w}}_{x}$:

(Equation 6) $${\hat{w}}_{x}=m_{1}R_{\text{choice}}+m_{2}PE_{\text{choice}}+m_{3}CC_{\text{choice}}+m_{4}R_{\text{out}}+m_{5}PE_{\text{out},Q}+m_{6}PE_{\text{out},V}+m_{7}CC_{\text{out}}$$

**Table 2:** Direction vector definitions

Choice				
	*V*_*block*_	*Q*_*ch*,*choice*_	*Q*_*unch*_	
**R**_**choice**_	0	1	0	–
**PE**_**choice**_	−0.5	0.5	0	–
**CC**_**choice**_	0	0.5	−0.5	–
Outcome				
	*V*_*trial*_	*Q*_*ch*,*out*_	*r*_*ch*_	*r*_*unch*_
**R**_**out**_	0	0	1	0
**PE**_**out,Q**_	0	−0.5	0.5	0
**PE**_**out,V**_	−0.5	0	0.5	0
**CC**_**out**_	0	0	0.5	−0.5

Direction vectors representing each of the predictions in Table 1, separately for choice and outcome predictors. Rows correspond to direction vectors, and columns correspond to dimensions, where each dimension represents the effect of an RL variable (e.g., *r*_*ch*_) on valence. Cells show the pattern of effects predicted by each vector; for example, **CC**_**out**_ predicts a positive effect of *r*_*ch*_ and an equal and opposite effect of *r*_*unch*_, while predicting no effects of other variables. Because the lengths of direction vectors are not fixed, the specific effect sizes displayed here are arbitrary; by convention, direction vectors have unit length, so we set effect sizes such that each vector had a length of 1 in Manhattan distance. To determine the actual lengths that should be assigned to these vectors, we used an optimization algorithm to identify the set of lengths which produced the best approximation to the observed RL variable effects (see Equation 6 and STAR Methods).

Using an optimization routine, we identified the set of non-negative lengths **m** that minimized the distance between the actual and approximate vector of effects ${\left\|w_{x}-{\hat{w}}_{x}\right\|}_{2}$. In the supplemental information, we report recovery analyses which show that this analysis method yields valid estimates of vector lengths (Method S2).

Notably, it might appear that our research questions could be addressed through a more straightforward analysis: the RL variables **x** in Equation 5 could be replaced with the variables defined in Table 1 (e.g., *CC*_*choice*_) to directly estimate the effects of these variables on affective valence. However, because these variables are conceptually confounded, the resulting effect estimates would not have the desired interpretation. For example, if *R*_*out*_ (*r*_*ch*_) and *PE*_*out*,*Q*_ (*r*_*ch*_-*Q*_*ch*_) were included in the same model, the weight associated with *PE*_*out*,__*Q*_ would not indicate the effect of reward prediction errors *r*_*ch*_-*Q*_*ch*_ on valence but merely the effect of expected reward *Q*_*ch*_, since it represents the effect of *r*_*ch*_-*Q*_*ch*_ when controlling for *r*_*ch*_. In the supplemental information (Method S1), we demonstrate that similar issues would arise with the interpretation of all effect estimates from this model, resulting in misleading conclusions. The present analysis circumvents these issues by estimating effects of conceptually distinct RL variables, and then identifying the combination of hypotheses that best accounts for the pattern of effects. This method allows us not only to assess specific predictions (e.g., *PE*_*out*,*Q*_) but also broader theories (e.g., the prediction error theory): vectors associated with the same theory can be summed so that each theory is represented by a single vector with a single length.

### Study 1 results

In the following, we report the lengths of vectors representing specific predictions and broader theories. More specifically, we report the ratio of each vector’s estimated length to the length of the RL variable effect vector **w**_**x**_. This ratio represents the portion of the RL variable effects explained by that prediction or theory. When describing posterior distributions for length ratios and other effects, we report the median, the probability of direction *pd* (i.e., the probability that the effect is in the direction of the median; *pd* > 95% was considered strong evidence of an effect),^45^ and the 95% credible interval (CI; the 2.5^th^ and 97.5^th^ percentiles of the posterior distribution).

**Figure 3:**
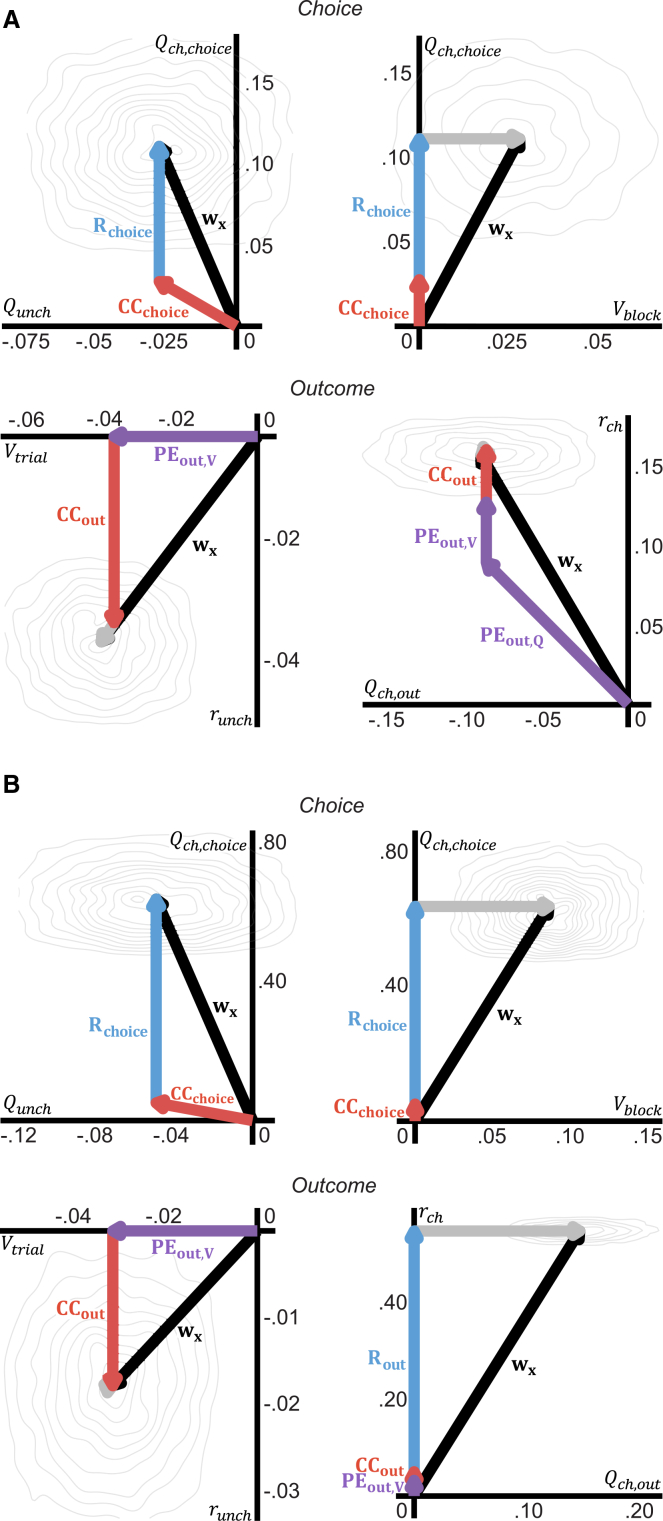
Effect vector decomposition These plots provide a geometric visualization of how the RL variable effect vector was decomposed into vectors representing theoretical predictions (Tables 1 and 2). (A) shows results for study 1, and (B) shows results for study 2. Because the full space of RL variable effects has seven dimensions, it cannot be displayed in a single plot. Instead, we show vectors projected into four 2D subspaces, where each subspace is defined by a pair of RL variable dimensions; collectively, the four plots display the 7 dimensions (with *Q*_*ch*,*choice*_ appearing twice). Within each subplot, the colored arrows represent the fitted direction vectors—more specifically, the vectors defined in Table 2 when scaled by their estimated (posterior mean) lengths. The black arrow represents the effect vector, and the gray arrow represents the unexplained residual. The gray contour lines represent uncertainty in the RL variable effect estimates (i.e., the posterior distribution of **w**_**x**_), which propagates to uncertainty in vector length estimates. The contour lines cover a 95% CI. For plotting, dimensions were rescaled to be roughly proportional to vector lengths along each dimension. Figure 4 provides more detailed information on the estimated length of each vector shown here. See also Figure S8.

**Figure 4:**
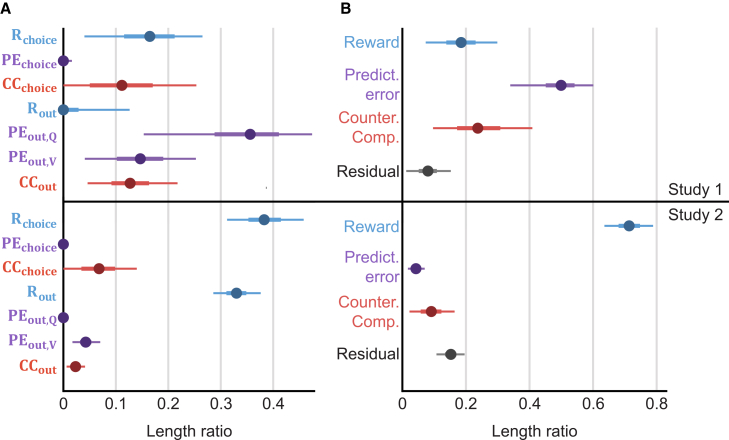
Vector length estimates (A) The lengths of vectors representing valence predictions. See Table 2 for vector definitions. (B) The lengths of vectors representing theories. “Residual” represents the portion of the effect vector not explained by these theories. “Predict.” is short for “prediction,” and “Counter. Comp.” for “counterfactual comparison.” Lengths were calculated in Manhattan distance, and then divided by the length of the RL variable effect vector, producing a “length ratio.” Dots represent the posterior median of the estimate; thick lines represent 50% CIs; thin lines represent 95% CIs. In each image, the estimates from study 1 are in the top half of the plot and estimates from study 2 are in the bottom half. See also Figures S1–S3 and S7 and Tables S3 and S4.

Predictions from all three theories accounted for unique portions of the RL variable effects (Figure 3A): **R**_**choice**_ (median: 0.16 [0.01, 0.28], *pd* = 97.9%), **PE**_**out**,**Q**_ (median: 0.36 [0.11, 0.49], *pd* = 99.4%), **PE**_**out**,**V**_ (median: 0.15 [0.02, 0.27], *pd* = 99.2%), and **CC**_**out**_ (median: 0.13 [0.03, 0.24], *pd* = 99.7%) all had positive lengths (Figure 4A). Correspondingly, the vectors for each theory had positive length (Figure 4B), indicating that all theories explain distinct portions of the effects (reward median: 0.18 [0.05, 0.33], *pd* = 99.8%; prediction error median: 0.50 [0.30, 0.62], *pd* > 99.9%; counterfactual comparison median: 0.24 [0.07, 0.44], *pd* > 99.9%). The posterior probability that the effects were best explained by a combination of all theories (i.e., that all theory vectors simultaneously had positive length) was 99.8% (Table S5).

To determine whether some theories had greater explanatory power than others, we compared the lengths of the theory-level vectors. The prediction error theory explained a greater portion of the RL variable effects than the reward theory (median difference: 0.32 [0.03, 0.49], *pd =* 98.1%). We did not find differences between the prediction error and counterfactual comparison theories (median difference: 0.26 [-0.10, 0.52], *pd =* 93.0%), or the reward and counterfactual comparison theories (median difference: 0.05 [-0.19, 0.35], *pd =* 63.2%)

### Study 2: Re-evaluating the determinants of affective valence in a new task

**Figure 5:**
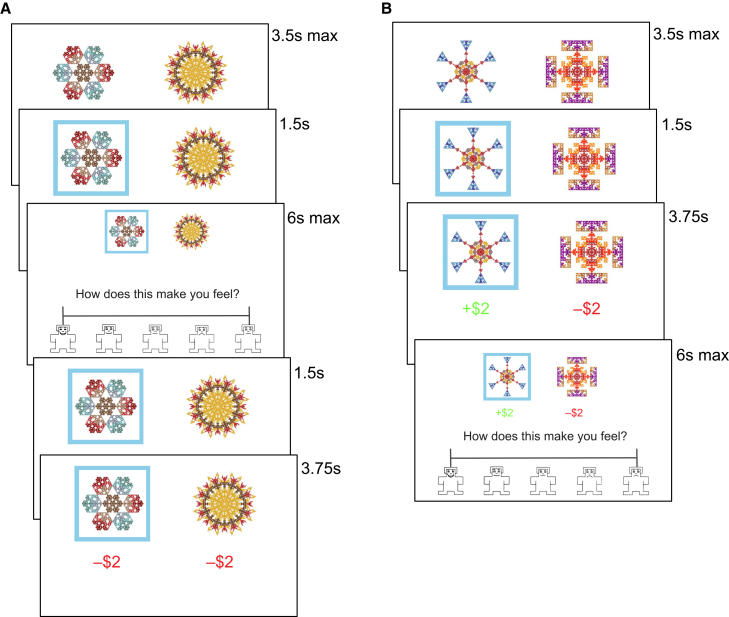
Study 2 task On each trial, participants were presented with a pair of cues, chose between them, and then viewed the outcome of each cue (a win or loss of $2). Participants completed two blocks of trials. In each block, two pairs of cues were presented 26 times each. (A) In one block, participants rated the valence of their affective response to the chosen cue. (B) In the other block, participants rated the valence of their response to the outcome of their choice. The order of these blocks was counterbalanced across participants.

In study 1, we found that reward, prediction errors, and counterfactual comparisons all made unique contributions to affective experience. In study 2, we sought to verify that this finding was robust to details of the task design. Thus, we collected a preregistered sample (*N* = 191) of a two-armed bandit task with valence self-reports that used alternative variants of the affect measure and RL task. Specifically, while in study 1 cues had fixed reward contingencies and yielded a variety of dollar amounts, in study 2 cues had drifting reward contingencies and outcomes were binary (either +$2 or -$2). Therefore, to learn which cues were most rewarding, study 2 participants had to track the recent percentage of wins rather than the long-term average of rewards. Additionally, study 2 participants directly reported the impacts of cues and outcomes on their affect (i.e., how cues and outcomes made them feel; Figure 5). By contrast, in study 1, the affective impacts of cues and outcomes were inferred from general affect ratings (Figure 2). Reports of general affect (study 1) and direct reports of affective responses (study 2) are both commonly used to assess the affect elicited by stimuli.

We predicted that, as in study 1, reward, prediction errors, and counterfactual comparisons would all influence affective responses. However, study 2 was not simply a replication of study 1: we expected that the affect measure used in study 2 could increase the influence of reward on reported valence, because the prompt directed participants’ attention toward the affective significance of what they chose or received—rather than to expectations or counterfactuals. Thus, study 2 allowed us both to verify that all three factors contribute to affective valence and to explore the different emphases placed on these factors by different self-report formats.

### Study 2 results

**Figure 6:**
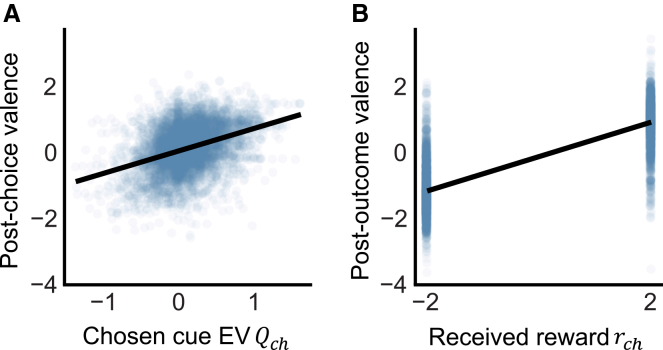
Study 2 valence data Valence ratings (*Z*-scored within-subject) plotted against key RL variables. Each dot represents a single rating. Black trend lines were estimated by regressing ratings onto the displayed variable (simple linear regression). (A) Post-choice valence ratings plotted against the expected value of the chosen cue *Q*_*ch*_. Posterior medians were used for *Q*_*ch*_ estimates. (B) Post-outcome valence ratings plotted against the (binary) reward from the chosen cue *r*_*ch*_.

We estimated the same effects of RL variables on affective valence **w**_**x**_ that were estimated in study 1, using a similar computational model (see STAR Methods; Figure 6). The model fit well: *R*^2^ = 0.44 for ratings made after choices, *R*^2^ = 0.86 for ratings made after outcomes (ratings were predicted by Equations 16 and 17). We then estimated the set of vector lengths **m** that best approximated **w**_**x**_ (Figure 3B). As in study 1, predictions from all theories accounted for unique portions of the RL variable effects: **R**_**choice**_ (median: 0.38 [0.30, 0.47], *pd* > 99.9%), **R**_**out**_ (median: 0.33 [0.28, 0.39], *pd* > 99.9%), **PE**_**out**,**V**_ (median: 0.04 [0.01, 0.07], *pd* = 99.7%), and **CC**_**out**_ (median: 0.02 [0.002, 0.04], *pd* = 98.6%) all had positive lengths (Figure 4A). Similarly, we found that vectors for the reward (median: 0.71 [0.62, 0.80], *pd* > 99.9%), prediction error (median: 0.04 [0.01, 0.07], *pd* = 99.7%), and counterfactual comparison (median: 0.09 [0.02, 0.18], *pd* = 99.9%) theories had positive lengths (Figure 4B), with a 99.6% posterior probability that the effects were best explained by the combination of all theories (Table S5). Thus, our results confirm that reward, prediction errors, and counterfactual comparisons all contribute to affective valence, and demonstrate that this finding is robust to variation in the RL task and affect measure.

### Comparing vector lengths across studies

As expected, the reward theory accounted for a larger portion of the RL variable effects in study 2 than it did in study 1 (median difference: 0.53 [0.36, 0.69], *pd* > 99.9%), with both of its predictions accounting for more of the effects (**R**_**choice**_ median difference: 0.22 [0.07, 0.38], *pd =* 99.9%; **R**_**out**_ median difference: 0.32 [0.17, 0.38], *pd >* 99.9%). Correspondingly, the portions explained by the prediction error (median difference: −0.46 [-0.58, -0.25], *pd* > 99.9%) and counterfactual comparison (median difference: −0.14 [-0.36, 0.04], *pd =* 93.2%) theories were smaller, with several of their predictions accounting for less of the effects (**PE**_**out**,**Q**_ median difference: −0.36 [-0.49, -0.10], *pd =* 99.4%; **PE**_**out**,**V**_ median difference: −0.10 [-0.24, 0.02], *pd =* 94.4%; **CC**_**out**_ median difference: −0.10 [-0.21, -0.01], *pd =* 98.1%). Indeed, the reward theory explained a greater portion of the RL variable effects in study 2 than did the prediction error or counterfactual comparison theories (median for comparison to prediction error: 0.67 [0.57, 0.77], *pd* > 99.9%; median for comparison to counterfactual comparison: 0.62 [0.45, 0.77], *pd* > 99.9%). These results suggest that common, alternative self-report formats might place meaningfully different emphases on the multiple determinants of affective valence.

### Investigating the role of affect in RL

After assessing the contributions of RL-related computations to affective valence, we next examined how affect—in turn—contributes to RL. As we discussed in the Introduction, standard RL models assume that behavior is reinforced by external reward (e.g., money), such that choice options which have typically led to greater reward are more likely to be selected. For example, this is assumed by *Q*-learning models, which use the history of reward from each option (i.e., reward associations *Q*) to predict choice. However, we hypothesized that affect also reinforces behavior, such that choice options which have typically led to more affectively positive outcomes are also more likely to be selected. To test this hypothesis, we fit new models that allowed both the history of affective responses and the history of reward to predict choices.

These new models were largely the same as the originals: they predicted valence ratings according to Equations 5, 16, and 17 and updated reward associations *Q* according to Equation 1. However, the new models incorporated an additional choice predictor: affect associations *A*. For each trial *t* on which cue *c* was chosen, the cue’s affect association *A* was updated as follows:

(Equation 7) $$A_{c,t+1}=A_{c,t}+\alpha \left(w_{x,\text{out}}\cdot x_{\text{out},t}-A_{c,t}\right)\;x_{\text{out},t}={\left[V_{trial,t},Q_{ch,out,t},r_{ch,t},r_{unch,t}\right]}^{⊤}$$

where **w**_**x**,**out**_ are the effects of **x**_**out**_ on valence estimated in Equations 5 and 17. Therefore, **w**_**x**,**out**_**·x**_**out**_ represents the affective response to the outcome (as estimated by our models), and *A*_*c*_ captures the (recency-weighted) history of affective responses following cue *c*. To estimate the effects of reward and affect associations on choice, we modeled the probability of choosing cue *a* over cue *b* as:

(Equation 8) $$p{\left(\text{choose}\;a\right)}_{t}=\sigma \left(\beta_{Q}\left(Q_{a,t}-Q_{b,t}\right)+\beta_{A}\left(A_{a,t}-A_{b,t}\right)+\beta_{S}\left(S_{a,t}-S_{b,t}\right)\right)$$

where *β*_*Q*_ and *β*_*A*_ are the effects of *Q* and *A* on choice, respectively. We standardized the differences between reward associations (*Q*_*a*,*t*_-*Q*_*b*,*t*_) and the differences between affect associations (*A*_*a*,*t*_-*A*_*b*,*t*_) (i.e., we divided them by their standard deviations) so that *β*_*Q*_ and *β*_*A*_ were on a common scale.

We fit this model to data from studies 1 and 2. If actions are reinforced by both affect and reward *per se*, then both affect and reward associations should have positive effects on choice likelihood, such that participants prefer cues that have previously led to more positive affect in addition to preferring those that have led to greater reward. The results confirmed our hypothesis: in each study, both affect and reward associations had positive effects on choice, indicating that behavior was reinforced by both affect and reward (*β*_*Q*_ study 1 median: 0.71 [0.55, 0.89], *pd* > 99.9%; *β*_*A*_ study 1 median: 0.20 [0.06, 0.35], *pd* = 99.9%; *β*_*Q*_ study 2 median: 1.59 [1.36, 1.86], *pd* > 99.9%; *β*_*A*_ study 2 median: 0.42 [0.29, 0.55], *pd* > 99.9%). Additionally, we found that reward associations had a stronger effect on choice than affect associations in both studies (study 1 median difference: 0.51 [0.24, 0.78], *pd* > 99.9%; study 2 median difference: 1.17 [0.89, 1.48], *pd* > 99.9%). In the supplemental information, we show that our results are robust to different modeling assumptions (Data S5), and report simulations showing that the effect of reward associations is not an artifact of noise in our estimates of affect (Data S6). Additionally, we show in Data S7 that the inclusion of *A* in these models improved their estimated out-of-sample predictive performance (though the improvement in study 1 was marginal, possibly due to overlaps between *Q* and *A* and limited *A* variance, discussed below).

These results provide a strong initial indication that affect reinforces choice alongside external reward, while also suggesting that reward may have a larger influence on choice. However, there are two important limitations to these analyses. First, studies 1 and 2 were designed to produce variance in reward associations, while variation in ffect associations was only incidental, meaning there was a bias toward finding a larger standardized effect of reward associations. Second, affective responses were a function of variables related to cue outcomes (e.g., chosen outcomes, unchosen outcomes, expected outcomes). Consequently, it is conceivable that *A* values predicted choice merely because they captured variance in participants’ reward associations not fully captured by *Q* values and not because affect reinforced behavior. Therefore, we designed a third study that allowed us to explicitly address this alternative explanation, and to provide a more unbiased comparison between the effects of affect and reward associations on choice.

### Study 3: Verifying and comparing the effects of reward and affect on choice

**Figure 7:**
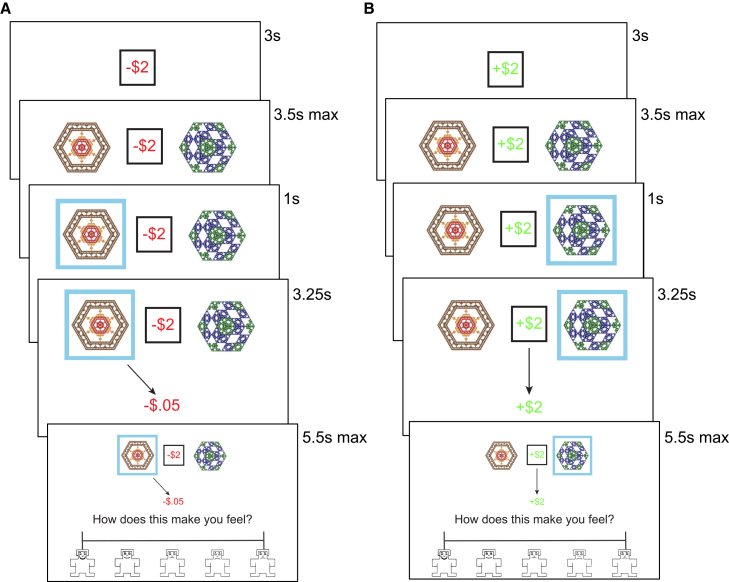
Study 3 task On each trial, participants were shown a “box amount” at the top of the screen (a win or loss of $2). They were then shown a pair of cues to choose between them. Three pairs of cues were presented 48 times each. On each trial, there was a 50% chance that the participant would receive the outcome of the chosen cue (A; cue outcomes were -$.05, $0, or +$.05), and a 50% chance that they would receive the box amount (B), irrespective of their choice. Participants also rated the valence of their affective response to the outcome of every trial. The range of box amounts (-$2 to $2) was set greater than the range of cue outcomes (-$.05 to +$.05) to maximize the influence of box amounts on affective responses, and thus to minimize the correlation between the valence elicited by cue outcomes and their reward values. In the supplemental information, we verify that learning and task engagement were as strong in study 3 as in studies 1 and 2, despite the use of smaller and noisier cue outcomes (Data S8).

We collected a third sample (*N* = 421) of a two-armed bandit task with valence self-reports (Figure 7). This study had two goals. First, we sought to confirm that both reward and affect associations influence choice in a preregistered, high-powered conceptual replication that optimally dissociated these two variables. Second, we sought to make an unbiased comparison between the effects of reward and affect associations on choice.

**Figure 8:**
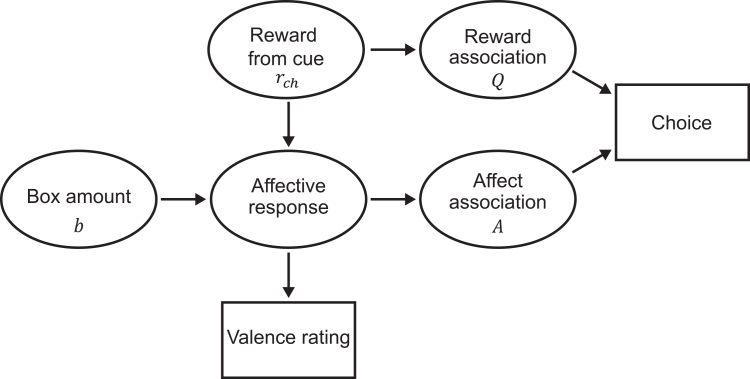
Study 3 key hypotheses A directed acyclic graph^46^ displaying key hypotheses from study 3. On trials where a cue outcome is received, the reward value of the outcome *r* and the box amount *b* contribute to the affective response to the outcome, which is reflected in valence ratings. Cues associated with more rewarding outcomes (i.e., with greater reward associations *Q*) and more positive affect (i.e., with greater affect associations *A*) are more likely to be chosen.

To dissociate the valence elicited by cue outcomes from their reward values, a monetary win or loss appeared in a box at the top of the screen at the start of each trial; we refer to these as “box amounts.” On every trial, there was a 50% chance that the participant would receive the outcome of the chosen cue, and a 50% chance that they would instead receive the box amount, meaning that these box amounts influenced expected reward. Given the effect of prediction errors on valence, we anticipated that greater box amounts would result in more negative affective responses to cue outcomes. Thus, box amounts should influence affective responses to cue outcomes without affecting their reward values, and therefore decorrelate reward and affect associations. Moreover, these box amounts provided a source of variance in affect that was fully independent from cue outcomes, which helped us to verify that affect associations influenced choice separately from reward associations (Figure 8). Notably, this design bears similarities to that of Hare and colleagues,^47^ who also manipulated prediction errors separately from the reward values of choice outcomes by presenting unrelated monetary amounts.

A second key feature of study 3 was that (unbeknownst to participants) reward contingencies were the same for all cues (each cue had an equal probability of yielding -$.05, $0, or +$.05), meaning that variation in reward associations was due only to the stochasticity of outcomes. This ensured that variation in reward associations was—like variation in effect associations—purely incidental, permitting an unbiased comparison of their effects on choice.

Importantly, participants were not aware that cues had the same reward contingencies; instead, they were instructed to learn which cues to choose through trial and error (see STAR Methods for more on the instructions). Behavioral analyses confirmed that the task elicited robust learning: the mean learning rate was higher in study 3 than in studies 1 and 2, and choices were comparably sensitive to past outcomes (Data S8). Thus, despite the equivalence of the underlying reward contingencies, study 3 was a learning task from the perspective of participants (like studies 1 and 2).

### Study 3 results

For trials on which the participant received an outcome from the chosen cue, valence ratings were modeled as follows:

(Equation 9) $$valence_{t}=w_{x}\cdot x_{t}+w_{z}\cdot z_{t\;}+\epsilon_{t}\;x_{t}={\left[r_{ch,t},b_{t},Q_{ch,t},V_{trial,t}\right]}^{⊤}$$

where *b* is the box amount (see STAR Methods for details). As predicted (Figure 8), box amounts had a negative effect on valence (median of standardized effect: −0.14 [-0.16,-0.12], *pd* > 99.9%) and the reward values of cue outcomes *r*_*ch*_ had a positive effect (median of standardized effect: 0.47 [0.44, 0.49], *pd* > 99.9%). The standardized effect of cue outcomes was somewhat stronger than that of box amounts (median difference of effect magnitudes: 0.33 [0.29, 0.36], *pd* > 99.9%), but the effect of box amounts was nonetheless strong enough to ensure that the correlation between the valence elicited by cue outcomes and their reward values was not excessively large (*r* = 0.61). Overall, the model predicted valence very well (*R*^2^ = 0.78).

For each trial on which the outcome of cue *c* was received, the cue’s reward association *Q* was updated according to Equation 1, and its affect association *A* was updated by:

(Equation 10) $$A_{c,t+1}=A_{c,t}+\alpha \left(w_{x}\cdot x_{t}-A_{c,t}\right)$$

where **w**_**x**_·**x**_*t*_ is the impact of the outcome on affective valence, as estimated by Equation 9. We predicted choices using *Q* and *A* values, as well as nuisance parameters that accounted for the influence of past trials on which the box amount was received (see STAR Methods). Replicating studies 1 and 2, we found that affect and reward associations both had positive effects on choice likelihood (*β*_*A*_ median: 0.51 [0.39, 0.64], *pd* > 99.9%; *β*_*Q*_ median: 0.29 [0.17, 0.41], *pd* > 99.9%), indicating that actions were reinforced by both affect and reward *per se*. In the supplemental information, we show that this result is robust to different modeling assumptions, and that the effect of reward associations is not an artifact of noise in our estimates of affect (as we did for studies 1 and 2; Data S5 and S6). Additionally, we show that the model including *A* has better out-of-sample predictive performance than an otherwise identical model that excludes this effect (Data S7).

Next, we considered a potential alternative explanation for this result. As we previously discussed, since affective responses are (partially) a function of variables related to cue outcomes (e.g., the chosen outcome, the expected outcome), it is conceivable that *A* values predicted choice merely because they captured variance in reward associations not captured by *Q* values. To address this possibility, we assessed whether two components of affective variance unrelated to cue outcomes predicted choice: box amounts and valence rating residuals.

We first considered valence rating residuals (i.e., variance not explained by our valence model; *ϵ*_*t*_ in Equation 9). If affect reinforces behavior, then even these residuals should predict choice, since they likely contain variance in affect not captured by our model (in addition to rating noise). On the other hand, if behavior is only reinforced by external reward, these residuals should have no predictive power, since they are unrelated to the reward values of cue outcomes (observed correlation: *r* < 0.001). To test the effect of valence residuals, we estimated the average residual valence *ϵ* elicited by outcomes from each cue *c* on recent trials *R*:

(Equation 11) $$R_{c,t+1}=R_{c,t}+\alpha \left(\epsilon_{t}-R_{c,t}\right)$$

We refer to this variable *R* as the “residual association.” We allowed residual associations *R* to predict choice likelihood alongside reward and affect associations (Equation 25). Participants were more likely to select cues associated with more positive residual valence (*β*_*R*_ median: 0.11 [0.08, 0.15], *pd* > 99.9%), supporting our conclusion that affect reinforced actions alongside reward.

Next, we tested the effect of box amounts on choice. Box amounts had a clear negative effect on affective responses to cue outcomes. Therefore, if affect reinforces behavior, cues whose outcomes are received in the presence of larger box amounts should be chosen less often. Conversely, box amounts were—by design—independent from the reward values of cue outcomes. Therefore, if behavior is reinforced by external reward alone, box amounts should have no effect on choice.

For each cue, we estimated the average box amount present on recent trials *t* where an outcome from that cue was received *M*:

(Equation 12) $$M_{c,t+1}=M_{c,t}+\alpha \left(b_{t}-M_{c,t}\right)$$

We refer to these values *M* as “box amount associations.” We estimated the effect of box amount associations on choice using a new model that was identical to the original model (Equation 25) except that *M* values were substituted for *A* values (including both *M* and *A* in the same model would have caused severe multicollinearity). Box amount associations had a negative effect on choice (*β*_*M*_ median: −0.13[-0.16, -0.09], *pd* > 99.9%): receiving a cue outcome in the presence of a larger box amount made the corresponding cue less likely to be chosen again. This result further confirms that behavior was reinforced by affect—not merely by external reward.

To further demonstrate how affect associations influenced task behavior, we next conducted an ablation analysis. We simulated 250 datasets from the posterior of the original study 3 model—the model which included effects of reward associations *Q* and affect associations *A* (Equation 25)—and another 250 datasets from an “ablated” model, in which the effect of affect associations *β*_*A*_ was fixed to 0 (see STAR Methods for details). We then assessed how well each set of simulations reproduced key effects observed in the actual data: in particular, participants’ tendency to prefer cues associated with better outcomes and worse box amounts.

We summarized this tendency using a simple model-free analysis (logistic regression): on trials where the cue outcome was received, we predicted whether participants would repeat their choice the next time the cue pair was presented (i.e., “stay” rather than “switch”) based on the outcome received *r*_*ch*_ and the box amount presented on the trial *b* (see STAR Methods). Consistent with our earlier modeling results, participants were more likely to repeat choices that led to better cue outcomes (*β*_*r*_ = 0.47 95% CI [0.44, 0.50], *p <* 0.001) and less likely to repeat choices made in the presence of larger box amounts (*β*_*b*_ = −0.07 95% CI [-0.10, -0.05], *p* < 0.001). The same regression was then applied to each simulated dataset. To capture the typical behavior predicted by each model, we computed the median effect estimates across the 250 simulations from the full model, and the median effect estimates across the 250 simulations from the ablated model.

**Figure 9:**
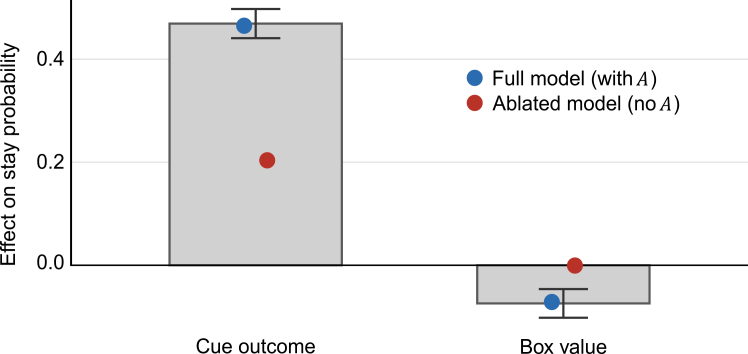
Impact of affect associations on choice behavior in study 3 On trials where the cue outcome was received, logistic regression was used to predict whether participants would repeat their choice the next time the cue pair was presented—i.e., “stay” rather than “switch”—based on the outcome received and the box amount present on the trial. Bars show the observed regression coefficients (log-odds) for these two predictors, with error bars representing 95% confidence intervals. To assess the relationship between affect associations and these effects, we simulated 250 datasets from the posterior of the full model—which includes an effect of affect associations (Equation 25)—and 250 datasets from an ablated model in which the effect of affect associations was fixed to 0. We applied the same logistic regression to each simulated dataset; the median effects across simulated datasets are shown as colored dots (blue: median effects from full model, red: median effects from ablated model). The full model closely reproduced both observed effects, validating its account of choice behavior. In contrast, the ablated model failed to capture the negative effect of box amounts and produced a substantially weaker effect of cue outcomes, demonstrating that affect associations account for a portion of both effects.

The results are shown in Figure 9. Simulations from the full model (blue dots)—which included an effect of affect associations *A*—reproduced the observed effects almost exactly, validating this model’s ability to capture the influence of both cue outcomes and box amounts on decision-making. Conversely, simulations from the ablated model (red dots) failed to account for the influence of box amounts on choice, demonstrating that this effect is uniquely explained by the impact of affect associations. Further, simulations from the ablated model produced a substantially attenuated effect of cue outcomes on choice, indicating that a considerable portion of this effect is also carried by affect associations. Altogether, these results illustrate the distinct and substantial contribution of affect associations to choice behavior.

Finally, we compared the effects of reward and affect associations on choice within the primary study 3 model (Equation 25). We found that affect associations *A* had a greater effect than reward associations *Q* in this task (median difference: 0.22 [0.001, 0.45], *pd* = 97.6%). This result further highlights the importance of affect to RL, demonstrating that the affective response to a decision outcome can be even more influential than its reward value.

## Discussion

Affective valence and RL are increasingly recognized to be closely connected, yet the exact nature of their relationship remains unclear. Here, we sought to illuminate this relationship by investigating the contributions of RL-related computations to affective valence and of affective valence to RL.

We began by considering existing theories of how RL computations determine valence. These theories often make overlapping predictions, such that evidence used to support one theory can typically be explained by others, leaving the true determinants of valence unclear. Here, we developed an original computational method to parse evidence for these overlapping accounts—one which conceptualizes theories as vectors in a space defined by RL variables. We applied this method in our studies, finding strong evidence that the effects of RL computations on affective experience are best explained by a combination of three leading perspectives: reward, prediction errors, and counterfactual comparisons all contribute to affective valence.

These results align with past findings that multiple different reward-related variables influence affective valence.^28^^,^^40^^,^^48^^,^^49^ However, due to the overlapping predictions of the theories considered here, it has typically been ambiguous whether such results support multiple theories—or which theories they support. For example, it has previously been found that both rewards and prediction errors influence affective valence during gambling tasks.^28^^,^^40^ This may be interpreted as evidence for both the reward and prediction error theories, but it has also been argued that these effects merely reflect two different forms of prediction error.^18^ To overcome this sort of ambiguity, we systematically quantified, separated, and tested the predictions of three leading perspectives. In doing so, we provide a novel empirical demonstration that the computations associated with all three views make distinct contributions to affective experience.

Furthermore, our findings indicate that two popular self-report formats have different sensitivities to the multiple determinants of affective valence. Specifically, we found a greater influence of reward on affective responses to cues and outcomes when participants directly reported their affective responses (Figure 5) than when these responses were inferred from ratings of general affect (Figure 2). The former self-report format may focus participants on the stimulus being rated (e.g., how winning $2 makes them feel) rather than comparisons to expectations or counterfactuals (e.g., how winning less than expected makes them feel) thus highlighting reward over other factors. Future research should further examine the different sensitivities of these common self-report variants, and the reasons for these differences, to inform their best use and interpretation (e.g., which format most accurately captures the relative importance of affective determinants?).

Altogether, these results provide a detailed picture of the determinants of affective valence. Future research could further develop this account by examining how these determinants relate to affective dimensions other than valence (e.g., arousal), or to specific emotions (e.g., fear and joy), as many of these constructs appear to have connections with RL as well.^35^^,^^50^ Indeed, the different RL computations considered here could plausibly correspond to different emotions (e.g., regret might depend on counterfactual comparisons while disappointment depends on prediction errors^28^). Conversely, future studies may consider how aspects of learning and decision-making not considered here may also contribute to affective experience. For example, the effort required to make a decision or the confidence a person has in their choice could easily influence their affective valence in ways the present results do not capture (e.g., mental effort often evokes negative affect,^51^ and decision confidence can modulate emotional responses to outcomes^52^). Thus, considering either a wider range of affective constructs or additional aspects of learning and decision-making could bring further insight into affective dynamics during RL. Beyond providing insights into the determinants of affect, our results also validate a novel computational framework for testing overlapping hypotheses. We show that this framework can effectively parse evidence for confounded predictions, integrate across predictions to quantify the explanatory power of general theories, and compare the power of predictions and theories within and across contexts. This analytic method represents a powerful tool for future research on affect and RL—and for other areas of research that contend with overlapping theoretical predictions.

After clarifying the contributions of RL-related computations to affective experience, we next examined the contributions of affect to RL. Standard RL models assume that behavior is reinforced by external reward, such that choice options which have typically led to greater reward (e.g., more money) are more likely to be selected. However, by modeling the determinants of choice across three RL tasks, we established that behavior is also reinforced by affect: participants preferred choice options that had previously led to more positive affect, in addition to preferring those that had led to greater reward. Moreover, we showed that affect can play an even larger role in reinforcing behavior than reward: affect associations predicted choice more strongly than reward associations in a two-armed bandit task (see “study 3 results”), suggesting that affective responses to decision outcomes can influence behavior even more strongly than their reward values. Altogether, these results expand upon traditional RL models by identifying an influential role for affective valence in shaping choice.

To illustrate this role, consider again an investor who earns modest returns from one stock during a market surge, and modest returns from another stock during a market downturn. The reward received from each stock is the same (a modest return), which will promote indifference in future choices between these (or similar) stocks. However, modest returns will likely make the investor feel worse (e.g., more disappointed) during a market surge than during a downturn, since expectations and counterfactuals are better during a surge. According to our model, this difference in valence will encourage the investor to prefer the stock held during the downturn. Thus, we argue that choices reflect the competing influences of reward and affect associations.

Affective responses often involve multiple interacting components, including stimulus evaluations (i.e., appraisals), physiological changes, action tendencies, expressions, and conscious experience.^53^ Each of these components can influence judgment and decision-making.^32^^,^^54^^,^^55^^,^^56^ Thus, additional research is needed to determine which forms of affective responding account for the influence of affect associations on choice.

Recognizing the multiple components of affective responses can also help to address a question that may arise for some readers: how can we know that choices were influenced by affective responses, and not merely by the computations which determined those responses? Past research has shown that decision outcomes are often evaluated relative to expectations and counterfactuals (i.e., that expectations and counterfactuals can serve as reference points^57^^,^^58^). Thus, our operationalizations of counterfactual comparisons and prediction errors may have captured reference-dependent value computations that both determined valence ratings and influenced future choices (e.g., perhaps study 3 participants evaluated cue outcomes relative to box amounts, and these relative valuations drove both affect ratings and subsequent decisions). However, valuations that determine affect qualify as appraisals,^35^^,^^59^^,^^60^ would necessarily manifest in conscious experience (if they impact valence ratings), and would likely also be expressed in physiology and other response components. Thus, for value computations that determine valence ratings to influence choice is for one or more components of affective responses (as we conceptualize them here) to influence choice. In other words, affect is best understood as potentially implementing certain value computations, rather than potentially being a byproduct of them.

This perspective raises another productive direction for future research: determining whether affect implements various reference-dependent value computations. For example, recent RL research indicates that outcome values are often encoded, in part, through a context-sensitive process that depends on rewards, counterfactuals, and expectations^61^^,^^62^; this valuation process could be a key mechanism of affective experience. Identifying valence with value computations like these would provide insight not only into affective experience but also into valuation and choice. For example, if it were found that affective valence encodes context-sensitive outcome values, this would suggest that factors which modulate valence (e.g., emotion regulation and emotional reactivity) also modulate the representation of these values and their effects on decision-making (e.g., since higher emotional reactivity leads to stronger affective responses, it might also lead to stronger effects of context-sensitive outcome values on choice). Future studies could measure and model valence (or other affective constructs) during RL tasks designed to capture context-sensitive outcome valuation^63^^,^^64^ to assess the connection between this processes and affect.

Our findings add to a growing body of literature on the functions of affect in RL. Past work has often emphasized the impact of a person’s affective state before receiving a decision outcome on how that outcome reinforces behavior.^18^^,^^19^^,^^65^ For example, Eldar and Niv^41^ found evidence that, in RL tasks, the valence of a person’s affect entering a trial can bias their valuation of subsequent choice outcomes (with more positive prior mood inducing more positive valuations). Here, we identify a complementary effect, demonstrating how the affective response to the choice outcome itself shapes future decisions.

Our results also dovetail with recent findings that social decisions are influenced by both affective valence and external reward.^7^^,^^33^ This has been demonstrated in tasks where participants are offered a certain quantity of money by a partner and are then given the option to punish their partner if they are unsatisfied with the offer (e.g., the Ultimatum Game^66^). It has been found that both the reward prediction error and the valence prediction error elicited by the offer (i.e., how much more or less money participants were offered than expected, and how much better or worse the offer made them feel than expected) influence punishment decisions. Similarly, our results imply that both reward and valence prediction errors update action valuations (i.e., cue preferences, as captured by *Q* and *A* values). Thus, our results confirm that reward and valence prediction errors influence subsequent decisions, but identify a different form that this influence can take: these prediction errors can shape the values of the actions that led to them, in addition to motivating actions toward the people who caused them.

Our account of affect and RL, and the potential elaborations of this account that we’ve discussed, stands to offer insight into a wide range of topics. For example, past research has shown not only that affective prediction errors influence social decisions, but that their impact on these decisions is blunted in depression, suggesting that affect-based RL could play an important role in both social learning and mental health.^7^ More broadly, by illuminating the determinants of valence and its role in RL, our results provide a foundation for better understanding the mechanisms underlying affective processes and traits (e.g., emotion regulation and affective pathology), as well as their effects on learning and choice. For example, identifying ways that emotion regulation techniques modulate the computations governing valence could clarify the mechanisms by which they impact affective experience. Similarly, our finding that affect reinforces choice suggests that blunted positive affect in response to rewards (e.g., in anhedonia) could impair the acquisition of appetitive behaviors, since attenuated affect should lead to attenuated reinforcement.

In summary, our results provide key insights into the bidirectional relationship between affective valence and RL. Moreover, they validate a powerful computational framework for testing overlapping hypotheses. Together, our findings and methods form a strong foundation for future research on affect-RL interactions, and on the implications of these interactions for diverse subjects.

### Limitations of the study

We highlight three limitations to our studies. First, we assessed affect using a unidimensional valence self-report scale. However, as discussed above, affective responses have several components (e.g., appraisal, physiology, and expression) and can be characterized along dimensions beyond valence (e.g., arousal and dominance) or in terms of specific emotions. These different forms and components of affect likely have somewhat differing relationships to RL (e.g., emotions of the same valence, such as sadness and frustration, may play different roles in learning^35^). Thus, achieving a fuller picture of the affect-RL relationship will require using a broader array of affect measures.

Second, our results identify multiple determinants of affective valence (reward, prediction errors, and counterfactual comparison) and of behavioral reinforcement (affect and reward), yet they do not clearly establish the relative importance of these variables. The contributions of different valence determinants and the impacts of affect and reward associations on choice varied considerably across studies, often in ways that appeared attributable merely to methodological decisions (e.g., the self-report format and the variance in average reward across cues). A related, third limitation is that our studies used a somewhat artificial learning task—an online two-armed bandit with small monetary rewards—which likely evoked affective responses of low intensity and complexity. Both of these limitations could be addressed by examining interactions between affect and RL in daily life, as recent research has begun to do.^67^^,^^68^^,^^69^ Such work could clarify the relative importance of the factors identified here for real-world affective experience and decision-making, and could verify our findings in a setting with greater ecological validity.

## Resource availability

### Lead contact

Requests for further information and resources should be directed to and will be fulfilled by the lead contact, Kevin LaBar (klabar@duke.edu).

### Materials availability

This study did not generate new unique materials.

### Data and code availability

• All study data have been deposited in the Duke Research Data Repository: https://doi.org/10.7924/r4r530 and are publicly available as of the date of publication.
• All original code has been deposited in the Duke Research Data Repository: https://doi.org/10.7924/r4r530 and is publicly available as of the date of publication.
• Any additional information required to reanalyze the data reported in this paper is available from the lead contact upon request.

## Acknowledgments

We thank Florence Wang for assistance with stimulus norming and task coding. This work was supported by a Graduate Grant Award from the Charles Lafitte Foundation Program for Research in Psychology & Neuroscience to D.F.P. and K.S.L.

## Author contributions

D.F.P., S.M.-K., G.R.S.-L., and K.S.L. designed the studies. D.F.P. and S.M.-K. designed the data analyses. D.F.P. coded and administered the experimental tasks and analyzed the data. D.F.P. drafted the manuscript. S.M.-K., G.R.S.-L., and K.S.L. provided edits and feedback.

## Declaration of interests

The authors declare no competing interests.

## Declaration of generative AI and AI-assisted technologies in the writing process

The authors used Claude (Anthropic) to assist with drafting and editing of select passages in the manuscript and supplemental information. The authors thoroughly reviewed and edited all text generated by Claude and take full responsibility for the content of the published article.

## STAR★Methods

### Key resources table

**Table:** 

REAGENT or RESOURCE	SOURCE	IDENTIFIER
**Deposited data**		

Behavioral data from the reported experiments.	This article	Duke Research Data Repository: https://doi.org/10.7924/r4r530

**Software and algorithms**		

R (version 4.3.2)	R core team^70^	https://www.r-project.org/
CmdStan (version 2.33.1)	Stan Development Team,^71^ Gabry et al.^72^	https://mc-stan.org/docs/cmdstan-guide/
PsychoPy (version 2021.2.3)	Peirce et al.^73^	https://www.psychopy.org/
Custom analysis code	This article	Duke Research Data Repository: https://doi.org/10.7924/r4r530

### Experimental model and study participant details

#### Study 1 participants

We collected data from 101 undergraduate students recruited from the subject pool of the Psychology & Neuroscience department. We excluded 22 participants for failing to meet data-quality criteria, resulting in a final sample of 79 (58% female, 41% male, 1% non-binary; *M*_age_ = 19.7, *SD*_age_ = 2.3). Participants were compensated with course credit and a bonus payment (equal to in-task earnings) of between $2 and $6.

#### Study 2 participants

We recruited 191 participants through Prolific Academic. All participants were adults, lived in the United States, and were fluent in English. We excluded 71 participants for failing to meet pre-registered data-quality criteria, resulting in a final sample of 120 (53% female, 42% male, 5% non-binary; *M*_age_ = 37.1, *SD*_age_ = 13.8). Participants were paid a base rate of $6 (approximately $10.50 per hour) and were also given a bonus payment (equal to in-task earnings) of between $1 and $5.

#### Study 3 participants

We recruited 421 undergraduate students recruited from the subject pool of the Psychology & Neuroscience department. We excluded 98 participants for failing to meet pre-registered data-quality criteria, resulting in a final sample of 323 (67% female, 33% male, 0% non-binary; *M*_age_ = 18.9, *SD*_age_ = 1.1). Participants were compensated with course credit and a bonus payment (equal to in-task earnings) of between $1 and $6.

#### Consent and approval

All study protocols were approved by the Duke University Institutional Review Board (protocol #2026-0503), and complied with all relevant ethical regulations. Informed consent was obtained from all participants.

#### Demographic statistics and analyses

We did not analyze the relationship between gender and the effects of interest because we did not have gender-related hypotheses, and because our studies were not designed or powered to reveal gender-related effects. Further, we do not report demographic variables beyond age and gender (e.g., ethnicity, SES) because no other variables were collected. The absence of results indicating how gender interacts with affective valence and RL, as well as the absence of certain demographic data, constitutes a limitation to the generalizability of our findings.

### Method details

#### Study format

All studies were completed online via the experiment platform Pavlovia. Experimental tasks were written in Psychopy 3.^73^ Data were recorded without direct identifiers.

#### Study 1 details

##### Sample size

Previous studies identified effects of reward-related variables on valence during learning and decision-making tasks using between 21 and 75 participants, with participants making between 9 and 66 valence ratings.^40^^,^^41^^,^^48^^,^^74^^,^^75^^,^^76^ Thus, we targeted a sample size at the top of this range. Participants completed 126 choice trials and 84 valence ratings. After excluding participants for poor data quality, our final sample size was 79 (data quality criteria are described below).

##### Procedure

Participants completed two blocks of a two-armed bandit task. In each block, three different cue pairs were presented 21 times each, in pseudorandom order. Participants were instructed that some cues gave better outcomes than others, on average, and that they could learn which cues to choose by paying attention to the outcomes. Cue outcomes were whole dollar amounts ranging from -$5 to +$5. The average outcome from each cue was between -$2.02 and +$2.02, and the standard deviations of the outcomes were between $2.49 and $3.44. Cues within each pair differed on average outcome but were matched on variance.

On one third of trials, valence and arousal rating prompts were presented after the cue outcomes were revealed; on another third of trials, a prompt was presented immediately after a choice was made; and on the remaining third of trials, no prompt was presented. The three trial types were pseudorandomly interleaved. Arousal data are not reported on here.

##### Data quality criteria

We excluded participants who met any of the following criteria:

• Failed multiple attention checks
• Answered three or more instruction comprehension questions incorrectly
• Did not make a choice within the time limit on more than 20% of trials, or on six or more trials consecutively
• Chose the cue on the left/right side of the screen on more than 80% of trials
• Skipped more than 14% of affect ratings

We also excluded participants who met any two of the following criteria:

• Failed one attention check
• Answered two instruction comprehension questions incorrectly
• Did not make a choice within the time limit on more than 10% of trials
• Skipped more than 7% of affect ratings
• Skipped a round of practice trials
• Did not respond appropriately on five or more consecutive instruction slides

Finally, we excluded an additional six subjects whose valence rating distributions showed no meaningful variation (e.g., nearly all ratings were at the neutral point). All exclusion decisions were made before data were analyzed.

##### Preregistration

Study 1, and related analyses, were not preregistered. Data were collected between March 25^th^ and April 20^th^ of 2022.

#### Study 2 details

##### Sample size

We used G∗Power^77^ to estimate the minimum sample necessary to achieve 80% power to detect positive lengths of all vectors assigned positive length in Study 1. We made the conservative assumption that vectors would be two-thirds the length they were in Study 1. This analysis indicated that 89 participants would provide sufficient power (further details of the power analysis are described in the preregistration; OSF: https://doi.org/10.17605/OSF.IO/F2PZY). Since these analyses were only approximate, we chose to oversample: after excluding participants who failed preregistered data quality criteria, our final sample was 120 (quality criteria are described below).

##### Procedure

Participants completed two blocks of a two-armed bandit task. In each block, two pairs of cues were presented 26 times each, in pseudorandom order. Cue outcomes were either +$2 or -$2. Each cue had a probability of yielding +$2 (as opposed to -$2) that drifted gradually throughout the task, with reflecting boundaries at 80% and 20%. Participants were informed that the likelihood of a cue yielding +$2 would gradually change over time, and that – on each trial – they should try to choose the cue that was currently more likely to lead to a win.

In one of the blocks, participants rated the valence of their affective response to the outcome at the end of every trial. In the other block, participants rated their affective response to the chosen cue immediately after making a choice. The order of these blocks was counterbalanced across participants.

##### Data quality criteria

We excluded participants who met any of the following criteria:

• Failed multiple attention checks
• Answered three or more instruction comprehension questions incorrectly
• Did not make a choice within the time limit on more than 20% of trials, or on six or more trials consecutively
• Chose the cue on the left/right side of the screen on more than 80% of trials
• Skipped more than 14% of valence ratings
• Standard deviation of valence ratings was less than 10% of the scale
• Standard deviation of valence ratings within a single block was less than 5% of the scale

We also excluded participants who met any two of the following criteria:

• Failed one attention check
• Answered two instruction comprehension questions incorrectly
• Did not make a choice within the time limit on more than 10% of trials
• Skipped more than 7% of valence ratings
• Skipped a round of practice trials
• Did not respond appropriately on five or more consecutive instruction slides

These criteria were preregistered.

##### Preregistration

We preregistered the task, data collection procedures, and computational model on OSF: https://doi.org/10.17605/OSF.IO/F2PZY. Additionally, we preregistered all valence predictions (Table 1) and the hypothesis that reward, prediction errors, and counterfactual comparisons would all contribute to valence. We did not preregister the use of vectors to test these predictions; however, we replicated all findings using our preregistered analysis approach (Data S9). We also did not preregister the hypothesis that affect associations would predict choice. The preregistration was submitted on June 10^th^, 2023; data were subsequently collected on June 10^th^ and June 11^th^.

#### Study 3 details

##### Sample size

We used G∗Power^77^ to estimate the minimum sample necessary to achieve 90% power to detect positive effects of reward associations *Q* and affect associations *A* on choice. This analysis indicated that 41 participants would provide sufficient power, assuming that effect sizes would be similar to those observed in pilot data (further details of the power analysis are described in the preregistration; OSF: https://doi.org/10.17605/OSF.IO/E6AJ7). Since our power analyses were highly approximate and data collection was inexpensive, we chose to oversample, collecting data throughout one full academic semester (end-of-semester stop rule). This process resulted in a sample of 323 participants who passed preregistered data quality criteria (criteria are described below).

##### Procedure

Participants completed three blocks of a two-armed bandit task. In each block, a different pair of cues was presented 48 times. Every cue had an equal probability of resulting in -$.05, $0, or +$.05. Participants were not told that all cues had the same reward contingencies. Instead, they were instructed that: “You must learn which shape to choose – and which to avoid – through trial and error…. On each trial, try to choose the shape that is currently better – the one that is currently giving better results, on average.”

On every trial, a value of +$2 or -$2 was shown in a box at the top the screen (the “box amount”). On one half of trials, participants received the box amount rather than the outcome of the chosen cue; on the other half of trials, they received the cue outcome. The trials on which the cue outcome was received were selected pseudorandomly. Participants were informed that, on each trial, there was a 50% chance of receiving the box amount, irrespective of their choice. At the end of every trial, participants rated the valence of their affective response to the outcome they received. The range of box amounts (-$2 to $2) was set greater than the range of cue outcomes (-$.05 to +$.05) to maximize the influence of box amounts on affective responses, and thus to minimize the correlation between the valence elicited by cue outcomes and their reward values.

Conceivably, the use of relatively small and noisy cue outcomes in this task could have disincentivized learning or reduced task engagement. We address this concern in the Supplemental Information by comparing learning rates, attention metrics, and the sensitivity of choices to past outcomes across the three studies. We show that learning and task engagement were as strong in Study 3 as in Studies 1 and 2 (and in some ways were stronger), resolving this concern (Data S8).

##### Data quality criteria

We excluded participants who met any of the following criteria:

• Failed multiple attention checks
• Answered 3 or more comprehension questions incorrectly
• Did not make a choice within the time limit on 10 or more choices consecutively
• Did not make a choice within the time limit on more than 20% of trials
• Skipped greater than 10% of valence ratings
• Standard deviation of valence ratings was less than 5% of the scale
• Chose the left/right cue on greater than 90% of the trials within a block
• Chose the left/right cue on greater than 85% of the trials within two blocks
• Chose the left/right cue on greater than 80% of all trials

We also excluded participants who met any two of the following criteria:

• Failed one attention check
• Answered 2 comprehension questions incorrectly
• Did not make a choice within the time limit on more than 15% of trials
• Skipped greater than 5% of valence ratings
• Standard deviation of valence ratings was less than 10% of the scale
• Chose the left/right cue on greater than 75% of trials

These criteria were preregistered.

##### Preregistration

We preregistered the task, data collection procedures, and computational model on OSF: https://doi.org/10.17605/OSF.IO/E6AJ7. Additionally, we preregistered the hypothesis that both reward and affect associations would influence choice. Our method for estimating the affective impacts of outcomes differed from the preregistered method; however, we replicated our findings using the preregistered method (Data S9). The preregistration was submitted on September 10^th^, 2023, and data were collected from September 11^th^ through November 27^th^, 2023.

#### Affect ratings

In all tasks, affect ratings were made using the Self-Assessment Manikin^78^ (shown in Figures 3, 6, and 7).

### Quantification and statistical analyses

#### Computational models

Here, we summarize the hierarchical Bayesian models used in each study. These models were selected from among alternatives using model comparison procedures described in the supplemental information (Data S1 and S2). The estimated values of model parameters not reported in the Results are shown in Table S6.

##### Study 1 initial model

We begin by describing the Study 1 model used to analyze the determinants of valence; this model was used to generate the results reported under “Study 1 results” and “Comparing vector lengths across studies.” As described in the Results, this model estimated reward associations *Q*– i.e., the expected reward from each cue – using a standard *Q*-learning algorithm (Equation 1). Additionally, to capture the possibility that participants partially “forgot” the values of cues that were not presented, we assumed that the reward associations of unshown cues *u* decayed toward 0:

(Equation 13) $$Q_{u,t+1}=Q_{u,t}+\alpha \left(0-Q_{u,t}\right)$$

where *α* is the learning rate from Equation 1. Model comparison indicated that this model was preferable to an alternative in which there was no decay, and an alternative in which the learning rate was replaced with an independent decay rate (Data S1).

We predicted choices using *Q*-values and a choice autocorrelation term *S* that captured participants’ tendency to repeat recent choices (Equation 2). On each trial, the *S* value for each presented cue *c* was updated by:

(Equation 14) $$S_{c,t+1}=S_{c,t}+\tau \left(s_{c,t}-S_{c,t}\right)$$

where *s*_*c*_ was 1 if *c* was chosen on *t* and 0 if it was not, and *τ* is a free parameter constrained to between 0 and 1.

Valence ratings were predicted from a set of seven RL variables whose effects were assumed to decay exponentially across trials (Equation 5). We list and define each of these RL variables in the Results sub-section, “study 1: exploring the determinants of affective valence.” For post-choice ratings, the values of *r*_*ch*_ (the reward from the chosen cue), *r*_*unch*_ (the reward from the unchosen cue), and *V*_*trial*_ (the expected value of the trial given the cue outcomes) on the current trial were set to 0, since these variables were only hypothesized to influence affective responses to outcomes. Because *Q*_*ch*_ (the expected value of the chosen cue) was hypothesized to influence affective responses to both choices and outcomes, we estimated effects of two different variables: *Q*_*ch*,*out*_, whose value on the current trial was set to 0 for post-choice ratings, and *Q*_*ch*,*choice*_, whose value was not set to 0 for these ratings. Valence ratings were z-scored within-participant before model fitting to prevent participants with greater rating variability from disproportionately impacting the results.

##### Study 2 initial model

Here, we describe the Study 2 model used to analyze the determinants of valence; this model was used to generate the results described under “Study 2 Results” and “Comparing vector lengths across studies.” Like the initial Study 1 model, this model estimated reward associations *Q*(i.e., expected cue values) using a standard *Q*-learning algorithm (Equation 1). Notably, this algorithm is just as applicable to Study 2 as it was to Study 1: *Q*-learning is standardly used to model human bandit tasks that, like Study 2, employ binary outcomes or drifting reward contingencies.^79^^,^^80^

The Study 2 model also allowed the reward associations of cues not presented *u* to decay toward 0:

(Equation 15) $$Q_{u,t+1}=Q_{u,t}+\eta \left(0-Q_{u,t}\right)$$

where *η* is the decay rate – a free parameter constrained to between 0 and 1 (model comparison favored an independent decay rate in Study 2; Data S1). As in Study 1, choices were predicted using *Q*-values and choice autocorrelation *S* (Equation 2); *S* was again estimated by Equation 14.

In Study 2, participants rated their affective responses to specific stimuli: cues and outcomes. Therefore, our model assumed that ratings were only affected by variables related to the stimulus being rated. When participants rated their response to a cue, ratings were predicted by:

(Equation 16) $$valence_{t}=w_{x,\text{choice}}\cdot x_{\text{choice},t}+w_{z}\cdot z_{t\;}+\epsilon_{t}\;x_{\text{choice},t}={\left[V_{block,t},Q_{ch,choice,t},Q_{unch,t}\right]}^{⊤}$$

When participants rated their response to the outcome, ratings were predicted by:

(Equation 17) $$valence_{t}=w_{x,\text{out}}\cdot x_{\text{out},t}+w_{z}\cdot z_{t\;}+\epsilon_{t}\;x_{\text{out},t}={\left[V_{trial,t},Q_{ch,out,t},r_{ch,t},r_{unch,t}\right]}^{⊤}$$

Baseline valence, block number, trial number, rating type, and previous rating (i.e., an autoregressive term) were included as the nuisance predictors **z.** As in Study 1, valence ratings were z-scored within-participant before model fitting. In the supplemental information (Data S2), we show that this model provided a better fit to the Study 2 data than a model that allowed for exponentially decaying effects of cues and outcomes (like the Study 1 model; Equation 5).

##### Studies 1 and 2: Adding affect associations

After analyzing the influence of RL computations on affect, we assessed the role of affect in RL. To do this, we fit new models to Studies 1 and 2 that were identical to the initial models, but with a new choice predictor added: affect associations. As described in the Results, affect associations *A* were updated according to Equation 7. The affect associations of cues not presented *u* were allowed to decay toward 0, in the same manner as reward associations *Q*. Thus, in Study 1, affect associations decayed according to the learning rate *α*:

(Equation 18) $$A_{u,t+1}=A_{u,t}+\alpha \left(0-A_{u,t}\right)$$

In Study 2, affect associations decayed according to an independent decay rate *η*:

(Equation 19) $$A_{u,t+1}=A_{u,t}+\eta \left(0-A_{u,t}\right)$$

Choices were predicted using both *Q*-values and *A*-values, as well as choice autocorrelation *S* (Equation 8). Valence ratings were predicted by the same equations used in the initial models (Equations 5, 16, and 17).

##### Study 3 model

Here, we describe the model used to generate the main Study 3 results. As in Studies 1 and 2, this model estimated reward associations *Q* using a standard *Q*-learning algorithm (Equation 1).

On each trial, participants received either the cue outcome or the box amount; we will refer to these as “cue-outcome trials” and “box-amount trials.” On cue-outcome trials, valence was predicted according to Equation 9. On box-amount trials, valence was predicted by:

(Equation 20) $$valence_{t}=w_{p}\cdot p_{t}+w_{z}\cdot z_{t\;}+\epsilon_{t}\;p_{t}={\left[b_{t},Q_{ch,t},V_{trial,t}\right]}^{⊤}$$

Because counterfactual outcomes were not shown in Study 3, *V*_*trial*_ could not be calculated as in Equation 4; instead, it was estimated using *Q*-values^19^:

(Equation 21) $$V_{trial,t}=Q_{ch,t}\cdot p\left(\text{choice}|t\right)+Q_{unch,t}\left(1-p\left(\text{choice}|t\right)\right)$$

Baseline valence and previous valence rating (i.e., an autoregressive term) were included as the nuisance predictors **z**. As in Studies 1 and 2, valence ratings were z-scored within-participant before model fitting.

As described in the Results, on cue-outcome trials we updated three associations for the chosen cue: reward associations *Q* (Equation 1), affect associations *A* (Equation 10), and residual associations *R* (Equation 11). On box-amount trials, we updated three analogous associations for the chosen cue: *B*, *V*, and *E*. These were updated by the received box amount, by the affective impact of this box amount, and by the valence rating residual, respectively:

(Equation 22) $$B_{c,t+1}=B_{c,t}+\phi \left(b_{c,t}-B_{c,t}\right)$$

(Equation 23) $$V_{c,t+1}=V_{c,t}+\phi \left(w_{p}\cdot p_{t}-V_{c,t}\right)$$

(Equation 24) $$E_{c,t+1}=E_{c,t}+\phi \left(\epsilon_{t}-E_{c,t}\right)$$

where **w**_**p**_·**p**_*t*_ is drawn from Equation 20, and *ϕ* is a free learning rate for box-amount trials (constrained to between 0 and 1), distinct from the learning rate *α* used for cue-outcome trials.

On each trial, all associations that were not updated – that is, all associations for unchosen cues, and associations tied to the other trial type – were allowed to decay toward 0 (as in Equation 15). Separate decay rates were estimated for the two trial types: one rate *η* was used for *Q*, *A*, and *R* (the associations updated on cue-outcome trials), and a second rate *λ* was used for *B*, *V*, and *E* (the associations updated on box-amount trials). Choices were predicted from all six associations, a choice autocorrelation term *S* (updated by Equation 14), and a free side-bias parameter *κ* that allowed participants to have a bias toward choosing cues on the left *l* or right *r* side of the screen:

(Equation 25) $$p{\left(\text{choose}\;l\right)}_{t}=\sigma \left(\beta_{Q}\left(Q_{l,t}-Q_{r,t}\right)+\beta_{A}\left(A_{l,t}-A_{r,t}\right)+\beta_{R}\left(R_{l,t}-R_{r,t}\right)+\beta_{B}\left(B_{l,t}-B_{r,t}\right)+\beta_{V}\left(V_{l,t}-V_{r,t}\right)+\beta_{E}\left(E_{l,t}-E_{r,t}\right)+\beta_{S}\left(S_{l,t}-S_{r,t}\right)+\kappa \right)$$

In the Supplemental Information, we report parameter recovery analyses which verify that the effects of interest – *β*_*Q*_, *β*_*A*_, and *β*_*R*_ – were identifiable within this model (Method S4).

#### Model estimation

For each study, we integrated RL algorithms and valence predictions into a single Bayesian model, written in Stan.^71^ These models were fit to choice and rating data. Parameters were estimated using Markov Chain Monte Carlo (MCMC) sampling, implemented by the ‘sample’ function from the cmdstanr package.^72^

Model parameters were estimated hierarchically: each participant had their own parameter values, which were drawn from normal, population-level parameter distributions. We estimated the means and standard deviations of the population-level distributions, and used these distributions as the priors for the participant-level parameter estimates. The means and SDs of the population-level distributions were given normal, weakly informative priors (detailed in Table S7), with one exception – the population means for the RL variable effects **w**_**x**_ were fit using shrinkage priors (see the vector length estimation section for details). When reporting effect estimates in the results section, we use population mean parameter values. Likewise, vector lengths were estimated from the population means of the RL variable effects **w**_**x**_. Residual variance in valence ratings was estimated at the population level only (with separate variances estimated for post-decision and post-outcome ratings in Studies 1 and 2). We could assume that residual variance would be roughly homogenous across participants since valence ratings were z-scored within-participant before being entered into the models. We applied the prior distribution *N*(0, 2) – truncated to exclude negative values – to all residual variance parameters.

For each model, we generated at least 4,000 posterior draws using four Markov chains. All models passed standard diagnostics, indicating that the distributions of draws adequately approximated the true posterior distributions: there were no divergent transitions, all sampling chains had an Energy-Bayesian Fraction of Missing Information (E-BFMI) of at least .2, and all parameters had a potential scale reduction statistic $\hat{R}$ of less than 1.05 and bulk and tail effective sample sizes of at least 400 (see the Stan Reference Manual^71^ for details on each index).

#### Vector length estimation

After fitting computational models to Studies 1 and 2, we estimated vector lengths using the posterior distributions generated by these models. More specifically, for each study, we assigned lengths to direction vectors (Table 2) based on the posterior distribution of the RL variable effects **w**_**x**_**.** For each draw from this posterior, we used the L-BFGS-B optimization algorithm,^81^ implemented in R's 'optim' function,^70^ to identify the set of non-negative vector lengths that produced the best approximation to the draw (Equation 6). Lengths were bounded below at 0 (inclusive), since length is a magnitude and thus should not be negative. Applying this optimization to every posterior draw yielded a posterior distribution for each vector’s length. In Method S3, we verify that this procedure reliably recovers the correct vector lengths from posterior draws of **w**_**x**_: it assigns appropriately positive length to vectors that account for portions of the RL variable effects, and precisely 0 length (not merely small length, but zero length) to vectors that do not.

To construct posterior distributions for theory-level vectors, we summed the fitted vectors for each theory (e.g., for the reward theory, we summed **R**_**choice**_ and **R**_**out**_ after their lengths had been assigned) and calculated the length of the resulting sum on each posterior draw. To determine the probability that the observed effects were best explained by a particular combination of theories, we calculated the percentage of posterior draws on which the lengths of those theory-level vectors – and only those vectors – were positive.

##### Multiple comparisons

Estimating the lengths of several vectors inflates statistical risk through multiple comparisons. To address this problem, our computational models applied a shrinkage prior to the effects of the RL variables on valence. Specifically, the prior distribution for the population means of these effects was *N*(0,*σ*), with *σ* estimated as a free parameter. This prior resolves the multiple comparisons problem for statistical tests based on the estimated effects (e.g., tests of vector lengths) by modeling the effects within a multilevel framework.^82^^,^^83^ In all models, the prior for the parameter *σ* was set to *N*(0, 1), and the priors for the population standard deviations of the RL variable effects were set to *N*(0, 2); these distributions were truncated to exclude negative values.

#### Statistics

Statistical details for each effect of interest (including both model parameters and vector lengths) are reported in the Results and Figures. To describe each effect, we calculated the median of its posterior distribution, a 95% credible interval (the 2.5th and 97.5th percentiles of the posterior distribution), and the probability of direction *pd* (the posterior probability that the effect was in the direction of the posterior median). To compare two effects, we calculated the difference between their posterior distributions, and then reported the median, 95% credible interval, and *pd* of this difference. A *pd* of greater than 95% was considered strong evidence of an effect (or difference).^45^ Since all effects within each model were estimated jointly, the relevant sample size for each estimate is that of the broader study; study sample sizes are described under “Model details” (above).

For the reported effect estimates to be trustworthy, posterior distributions must be sampled adequately, and the models' assumptions must be reasonably consistent with the data. We verified adequate posterior sampling using standard MCMC diagnostics, as described in the “model estimation” section. We assessed the fit of our models' assumptions to the data in two complementary ways. First, we used posterior predictive checks, comparing key features of the observed data to those of data simulated from the fitted models (Figures 3C, 3D, and 9). Second, we conducted model comparisons (Data S1 and S2), which evaluated whether our models captured the structure of the data better than alternatives making different assumptions. In the Supplemental Information, we also report a number of analyses demonstrating the robustness of our results to different modeling assumptions.

#### Ablation analyses

To conduct the ablation analyses reported in “Study 3 results,” we drew 250 sets of parameter values from the estimated posterior distribution of the Study 3 model. We simulated two datasets from each draw: one using the drawn parameter values as-is (the “full” model), and one using the same parameter values but with *β*_*A*_ fixed to 0 for all participants (the “ablated” model).

The same number of trials and subjects were used in the simulations as in the actual experiment. On each trial of a simulation, a choice and valence rating were generated according to the Study 3 model equations (Equations 9, 20, and 25); conversely, the outcome and box amount were fixed to those used on that trial in the actual experiment. At the end of each trial, reward, affect, and residual associations were updated (again, according to Study 3 model equations).

To assess the effects of cue outcomes and box values on simulated and actual behavior, we fit a logistic regression to each simulated dataset and to the real data. For each trial *t* on which a cue outcome was received, this regression model predicted whether the participant *p* would *stay* – i.e., repeat their choice – on the next trial based on the reward value of the cue outcome *r*_*ch*_ and the box value *b*:

(Equation 26) $$p\left(stay_{t+1}\right)=\sigma \left(\beta_{0,p}+\beta_{r}r_{ch,t}+\beta_{b}b_{t}\right)$$

where *σ*() is the logistic function. We excluded trials *t*+1 that were the first in the block, since, in these cases, the previous trial *t* involved a different cue pair. Note that this model was fit with subject fixed effects: that is, we fit a separate intercept to each participant’s data *β*_0,*p*_, but estimated the effects of interest (*β*_*r*_,*β*_*b*_) at the group level only. We chose this estimation method over a multi-level model to ensure fast and stable estimation across the large number of simulated datasets (500).
