# Supplementary material for: Investigating the relationship between affective valence and reinforcement learning
Source: iScience. 2026 Jul 22;29(8):116863. doi: 10.1016/j.isci.2026.116863 (PMC13427410; doi:10.1016/j.isci.2026.116863)
Supplement: Document S1. Figures S1–S8, Tables S1–S7, Data S1–S9, and Methods S1–S4 [file mmc1.pdf]

iScience, Volume *29*

Supplemental information

Investigating the relationship between affective valence and reinforcement learning

Daniel F. Parr, Seth Madlon-Kay, Gregory R. Samanez-Larkin, and Kevin S. LaBar

Data S1: Reinforcement learning model selection

For each study, we conducted a series of model comparisons to identify a reinforcement learning (RL) model that was appropriate for the data. We compared models using Pareto-Smoothed Importance Sampling Leave-One-Out Cross-Validation (PSIS-LOO-CV), which efficiently approximates pointwise leave-one-out cross-validation for Bayesian models [S1]. This method provides an estimate of model fit termed LOOIC (Leave-One-Out Information Criterion), which indicates how accurately the model can predict held out data (lower LOOIC values indicate better model fit). Notably, LOOIC has been shown to better approximate out-of-sample predictive accuracy than common alternatives such as WAIC, AIC, or DIC [S1]. Following other studies that have compared RL models using PSIS-LOO-CV [S2-S4], we used individual data points (i.e., choices and valence ratings) as the held out data, in order to maximize the precision and reliability of the LOOIC estimates.

Study 1

We began by fitting a simple Q-learning model with a softmax decision function, and then incrementally added features to capture nuisance variation. We tested the following features in the order listed:

- Choice autocorrelation: Subjects were assumed to maintain a value S for each cue that represented how often the cue was chosen on recent trials. For each cue c that was presented on trial t:
$$S_{c,t+1} = S_{c,t} + \tau\left(s_{c,t} - S_{c,t}\right) \quad (1)$$
where s_c is 1 if c was chosen and 0 if it was not chosen. S values were used to predict choice, as shown in Equation 2 (main article).
- Side bias: Subjects were allowed to have a bias κ toward choosing cues on the left or right side of the screen. When autocorrelation was also included, choice probabilities were predicted as:
$$p(\text{choose } l)_t = \sigma\left(\beta_Q\left(Q_{l,t} - Q_{r,t}\right) + \beta_S\left(S_{l,t} - S_{r,t}\right) + \kappa\right) \quad (2)$$
where l refers to the left cue and r to the right cue.
- Forgetting without independent decay rate: Q values of cues not presented u were decayed toward zero.
$$Q_{u,t+1} = Q_{u,t} + \alpha\left(0 - Q_{u,t}\right) \quad (3)$$
- Forgetting with independent decay rate: Q values of cues not presented u were decayed toward zero, with the decay rate fit as a free parameter η, rather than being set equal to α.
$$Q_{u,t+1} = Q_{u,t} + \eta\left(0 - Q_{u,t}\right) \quad (4)$$

Each time a new feature was added, we refit the model, and compared its LOOIC value to that of the previous model which lacked the feature. If the new model, with the added feature, had a LOOIC value of at least 8 points less than the previous model, and the difference was at least twice as large as the standard error of the difference, we considered this strong evidence that the new feature improved fit, and kept the feature in the model (consistent with recommendations made by Sivula, Magnusson, & Vehtari; [S5]). Otherwise, the feature was removed. The results of this procedure for Study 1 are shown in Table S1a.

Study 2

The same model selection procedure used in Study 1 was followed for Study 2. The results are shown in Table S1b.

Study 3

First, we fit a model that predicted choices using reward associations Q, affect associations A, and choice autocorrelation S. On each trial, the reward associations Q of unselected cues were allowed to decay toward 0 (Equation S4). Affect associations A were allowed to decay in the same manner:
$$A_{u,t+1} = A_{u,t} + \eta\left(0 - A_{u,t}\right) \quad (5)$$

This model allowed Q and A values to be updated on trials in which the box amount was received, in addition to trials in which the outcome of the cue was received. When the outcome of the cue was received, Q and A values were updated by:

$$Q_{c,t+1} = Q_{c,t} + \alpha\left(\beta_{Q,cue} \cdot r_{c,t} - Q_{c,t}\right) \quad (6)$$

$$A_{c,t+1} = A_{c,t} + \alpha\left(\beta_{A,cue} \cdot valence_t - A_{c,t}\right) \quad (7)$$

Table S1

a

Nuisance features	$LOOIC_{diff}$	SE_{diff}	$LOOIC_{diff}$ / SE_{diff}
None	0		
Autocorrelation	-1628.1	80.8	-20.2
Autocorrelation	0		
Autocorrelation, side bias	3.6	1.1	3.2
Autocorrelation	0		
Autocorrelation, forgetting with α	-54.5	20.3	-2.7
Autocorrelation, forgetting with α	0		
Autocorrelation, forgetting with η	-7.8	9.9	-0.8

b

Nuisance features	$LOOIC_{diff}$	SE_{diff}	$LOOIC_{diff}$ / SE_{diff}
None	0		
Autocorrelation	-1713.5	82.0	-20.9
Autocorrelation	0		
Autocorrelation, side bias	-5.7	6.9	-0.8
Autocorrelation	0		
Autocorrelation, forgetting with α	-62.5	35.9	-1.7
Autocorrelation	0		
Autocorrelation, forgetting with η	-101.1	24.3	-4.2

c

Choice predictors	$LOOIC_{diff}$	SE_{diff}	$LOOIC_{diff}$ / SE_{diff}
Q, A	0		
Q, A, S	-2359.2	99.4	-23.7
Q, A, S	0		
Q, A, S, B, V	-106	41.0	-2.6
Q, A, S, B, V	0		
Q, A, S, B, V, κ	-320.1	37.0	-8.6

RL model selection, related to STAR Methods. Each table shows the results from fitting and comparing a series of RL models, incorporating different sets of nuisance features, to data from each study. Panel A shows results for Study 1, Panel B for Study 2, and Panel C for Study 3. $LOOIC_{diff}$ is the difference

between the LOOIC of the current model and the model at the top of the subsection; SE_{diff} is the standard error of this difference; $LOOIC_{diff}$ / SE_{diff} is the ratio between the two.

When the box amount was received, Q and A values were updated by:

$$Q_{c,t+1} = Q_{c,t} + \alpha(\beta_{Q,box} \cdot b_{c,t} - Q_{c,t}) \tag{8}$$

$$A_{c,t+1} = A_{c,t} + \alpha(\beta_{A,box} \cdot valence_t - A_{c,t}) \tag{9}$$

where b is the box amount and $valence$ is the valence rating. Choices were predicted by:

$$p(\text{choose } a)_t = \sigma\left((Q_{a,t} - Q_{b,t}) + (A_{a,t} - A_{b,t}) + \beta_S(S_{a,t} - S_{b,t})\right) \tag{10}$$

To verify that the choice autocorrelation term S should be included in the model, we compared this model to one that did not include an effect of autocorrelation. The model that included S fit substantially better (Table S1c).

Next, we fit a model that assumed Q and A were only updated on trials in which the outcome of the cue was received, according to the following equations:

$$Q_{c,t+1} = Q_{c,t} + \alpha(r_{c,t} - Q_{c,t}) \tag{11}$$

$$A_{c,t+1} = A_{c,t} + \alpha(valence_t - A_{c,t}) \tag{12}$$

On trials in which the box amount was received, separate reward associations B and affect associations V were updated:

$$B_{c,t+1} = B_{c,t} + \phi(b_{c,t} - B_{c,t}) \tag{13}$$

$$V_{c,t+1} = V_{c,t} + \phi(valence_t - V_{c,t}) \tag{14}$$

These values were updated with a learning rate ϕ that was independent from the learning rate α, and were decayed toward 0 using a decay rate λ that was independent from the decay rate η. All of these associations were used to predict choice:

$$p(\text{choose } a)_t = \sigma\Big(\beta_Q(Q_{a,t} - Q_{b,t}) + \beta_A(A_{a,t} - A_{b,t}) + \beta_S(S_{a,t} - S_{b,t}) + \beta_B(B_{a,t} - B_{b,t}) + \beta_V(V_{a,t} - V_{b,t})\Big) \tag{15}$$

This model fit the data better than the initial model (Table S1c).

Finally, we added a parameter κ to this model that allowed participants to have a bias toward choosing cues on the left side of the screen (as in Equation S2). This model provided the best fit to our data (Table S1c). Notably, this model uses raw valence ratings to update A, while the model we report in the main article uses model-predicted valence to update A, and allows residual valence to have a separate effect on choice. The reasons for this discrepancy are explained under "Divergences from preregistration," below.

Data S2: Affect model selection

Study 1

To predict valence ratings in Study 1, we assumed that the effects of RL variables decayed exponentially across trials (Equation 5). Here, we report tests of two alternative assumptions. First, we fit a model that assumed effects decay every time step - rather than every trial - where there are two time steps per trial: the first time step corresponds to choosing a cue, and the second to viewing the outcome. The model was:

$$valence_{ts} = \mathbf{w_x} \cdot \sum_{i=1}^{ts} \gamma^{ts-i}\mathbf{x}_i + \mathbf{w_z} \cdot \mathbf{z}_{ts} + \epsilon_t \tag{16}$$

Second, we fit a model that assumed ratings were only influenced by the values of the RL variables on the current time step, with no effects across trials:

$$valence_{ts} = \mathbf{w_x} \cdot \mathbf{x}_{ts} + \mathbf{w_z} \cdot \mathbf{z}_{ts} + \epsilon_{ts} \tag{17}$$

We compared these models to the model that assumed effects decayed exponentially across trials, finding that the latter model provided the best fit (Table S2a).

Additionally, we tested an alternative formulation of V_{block} in which the learning rate was a free parameter θ, rather than being set equal to the learning rate for Q values α (as in Equation S3) :

$$V_{block,t} = V_{block,t-1} + \theta(r_{ch,t-1} - V_{block,t-1}) \tag{18}$$

We failed to find evidence that the addition of θ improved fit (Table S2b; standards of evidence are described under "Reinforcement learning model selection"). Thus, we used Equation 3 to calculate V_{block} in Study 1. For consistency between studies, we also used this formulation in Study 2.

Table S2

a

Model	$LOOIC_{diff}$	SE_{diff}	$LOOIC_{diff}$ / SE_{diff}
Decay across trials	0		
Decay across t.s.	38.2	14.3	2.7
No effects across t.s.	449.3	48.8	9.2

b

V_{block} learn. rate	$LOOIC_{diff}$	SE_{diff}	$LOOIC_{diff}$ / SE_{diff}
α	0		
θ	-3.9	8.4	-0.5

c

Model	$LOOIC_{diff}$	SE_{diff}	$LOOIC_{diff}$ / SE_{diff}
No effects across t.s.	0		
Decay across trials	654.8	84	7.8

Affect model comparisons, related to STAR Methods. Comparisons of alternative models for predicting valence ratings in Studies 1 and 2. $LOOIC_{diff}$ is the difference between the LOOIC of the current model and the best-fitting model, which is shown at the top of the table. SE_{diff} is the standard error of this difference, and $LOOIC_{diff}$ / SE_{diff} is the ratio of the two. **a.** In Study 1, a model that assumes the effects of RL variables on valence decay across trials fits best. **b.** In Study 1, calculating V_{block} using a free parameter θ for the learning rate does not improve model fit. **c.** In Study 2, a model that assumes RL variables only influence valence ratings on the current time step fits better than a model which assumes their effects gradually decay across trials.

Study 2

In Study 2, we assumed that valence ratings were only influenced by the values of RL variables on the current time step (Equations 16, 17). To ensure that this was an appropriate assumption, we compared this model to a model that assumed the effects of RL variables on valence gradually decayed across trials (Equation 5). The former model fit better, confirming our assumption (Table S2c).

Method S1: Interpreting effect estimates from Equation 5

In the main article, we note that if the RL variables $\mathbf{x}$ in Equation 5 were replaced with the variables defined in Table 1, the effect estimates associated with these variables would not have the desired interpretation. More specifically, suppose we fit the following model:

$$valence_t = \boldsymbol{\omega}_{\mathbf{v}} \cdot \sum_{i=1}^{t} \gamma^{t-i} \mathbf{v}_i + \boldsymbol{\omega}_{\mathbf{z}} \cdot \mathbf{z}_t + \epsilon_t$$
$$\mathbf{v_i} = \left[R_{choice,i}, PE_{choice,i}, CC_{choice,i}, R_{out,i}, PE_{out,Q,i}, PE_{out,V,i}, CC_{out,i}\right]^{\top} \quad (19)$$

Because the definitions of the variables $\mathbf{v}$ overlap, the weight $\boldsymbol{\omega}$ associated with a given variable would not represent the effect of that variable on valence, but instead would represent some combination of RL variable effects. Specifically, the weights $\boldsymbol{\omega}$ would relate to the RL variable effects $\mathbf{w}$ (as estimated via Equation 5) as follows:

$$\boldsymbol{\omega}[R_{choice}] = \mathbf{w}[V_{block}] + \mathbf{w}[Q_{ch,choice}] + \mathbf{w}[Q_{unch}]$$
$$\boldsymbol{\omega}[PE_{choice}] = -\mathbf{w}[V_{block}]$$
$$\boldsymbol{\omega}[CC_{choice}] = -\mathbf{w}[Q_{unch}]$$
$$\boldsymbol{\omega}[R_{out}] = \mathbf{w}[V_{trial}] + \mathbf{w}[Q_{ch,out}] + \mathbf{w}[r_{ch}] + \mathbf{w}[r_{unch}]$$
$$\boldsymbol{\omega}[PE_{out,Q}] = -\mathbf{w}[Q_{ch,out}]$$
$$\boldsymbol{\omega}[PE_{out,V}] = -\mathbf{w}[V_{trial}] \quad (20)$$

$$\boldsymbol{\omega}[CC_{out}] = -\mathbf{w}[r_{unch}]$$

Because the weights $\boldsymbol{\omega}$ cannot be interpreted in a straightforward manner (i.e., as the effect of the associated variable), they would not directly address our research questions.

To verify the above equalities, we simulated 1,000 datasets from the prior distribution of the hierarchical Bayesian model fit to Study 1, and fit Equation 5 and Equation S19 to each dataset. For the sake of computational efficiency, we did not estimate all variables in these equations from the simulated data. Instead, we fixed the values of most variables to the values used in the simulation, except for the weights $\mathbf{w}$ (in Equation 5) and $\boldsymbol{\omega}$ (in Equation S19). This allowed us to estimate these weights through simple linear regression (see analysis code for details: https://doi.org/10.7924/r4r530). All equalities in Equation S20 held across all 1,000 simulations, verifying their accuracy.

Illustrative simulation: aggregated variable analysis vs. vector-based analysis

The above demonstrates why the effect estimates from Equation S19 cannot be interpreted as intended. To make the advantages of the vector-based approach more concrete, we next provide an example in which attempting to interpret these effect estimates in a straightforward manner would lead to misleading conclusions, while our vector-based approach gives coherent results. In this section, we will refer to the analysis approach in which affect is predicted using the variables defined in Table 1 (e.g., CC_{out}) – as in Equation S19 – rather than the RL variables (e.g., r_{ch}, r_{unch}) – as in Equation 5 – as an "aggregated variable approach." The issues with the aggregated variable approach are especially acute when some RL variable effects contradict theoretical predictions, so we examined such a case for this illustration. The results of our examination are summarized in Figure S1: in a case where the aggregated variable approach yields incorrect effect estimates, the vector-based approach yields appropriate results. Below, we describe the analyses conducted for this illustration in detail.

We considered a simplified scenario in which only three RL variables impacted affective valence, all at the time of outcome: the reward received r_{ch}, the expected value of the chosen cue $Q_{ch,out}$, and the outcome of the unchosen cue r_{unch}. We assumed that reward had a positive effect on valence, but that prediction errors and counterfactual comparisons did not contribute. We chose a population mean of 0.5 for the effect of reward r_{ch}, with an SD of 0.25. This choice ensured a strong effect of reward that would allow for clear recovery results while still permitting substantial heterogeneity across subjects; additionally, this choice aligned the assumptions of this analysis with those of the analysis reported in the section "Vector length recovery analysis." In addition to the effect of r_{ch}, we assumed that $Q_{ch,out}$ and r_{unch} each had modest positive effects on affective responses to outcomes (population means: 0.25, SDs: 0.125) – effects not predicted to be positive by any theory. Such a scenario is plausible: some past studies have found positive effects of expected value on affective valence following outcomes [S6-S7], and counterfactual outcomes have sometimes been found to have positive effects on affect, such as when they make a person more optimistic about future possibilities [S8]. We even observed positive effects of certain forms of expected value in our own data (Figure S8). Moreover, even when effects of expectations or counterfactuals are not conclusively positive, a substantial fraction of posterior draws for these effects may nonetheless be positive – meaning that the dynamics illustrated here can still influence estimates in these cases.

We simulated 200 datasets using the trial structure from Study 1, under the assumption that data were generated according to the Study 1 model (see "Study 1: initial model" in the Methods). For each simulated subject, values of parameters other than the RL variable effects (e.g., learning rates, decay rates, baseline valence) were drawn from normal distributions whose means and SDs were set to the corresponding estimates from the fitted Study 1 model. Following Equation 5, we generated valence ratings as a linear function of the exponentially decayed sums of r_{ch}, $Q_{ch,out}$, and r_{unch}, plus nuisance terms and residual noise. For computational efficiency, we saved the exponentially decayed sums used to generate the data and provided them directly to the recovery analyses, rather than re-estimating the RL variables and sums from the simulated choices and outcomes (which would require fitting a computational model to each simulated dataset).

We analyzed each simulated dataset in two ways. Both of these analyses only considered the predictors R_{out}, $PE_{out,Q}$, and CC_{out} (Table 1), since in our simplified example only the RL variables related to these predictors (r_{ch}, $Q_{ch,out}$, and r_{unch}) were allowed to influence valence. First, we used the aggregated variable approach: we regressed valence ratings onto the exponentially decayed sums of R_{out} (= r_{ch}), $PE_{out,Q}$ (= $r_{ch} - Q_{ch,out}$), and CC_{out} (= $r_{ch} - r_{unch}$), along with nuisance terms. Second, we

used the vector-based approach: we regressed valence ratings onto the exponentially decayed sums of the three relevant RL variables (r_{ch}, $Q_{ch,out}$, and r_{unch}), and then identified the set of non-negative lengths for the $\mathbf{R_{out}}$, $\mathbf{PE_{out,Q}}$, and $\mathbf{CC_{out}}$ vectors (Table 2) that optimally approximated these effects. In both analyses, we used linear regression with subject fixed effects rather than hierarchical Bayesian estimation for computational efficiency.

The results are shown in Figure S1. The aggregated variable approach yielded clearly misleading estimates. The median recovered weight on $R_{out}$was approximately 1 – twice the true effect of reward (0.5). This occurred because the weight on R_{out} reflects the sum of all three RL variable effects (Equation S20), not the effect of reward alone. Meanwhile, the median recovered weights on $PE_{out,Q}$ and CC_{out} were approximately -0.25, falsely suggesting that prediction errors and counterfactual comparisons had negative effects on valence, even though neither variable influenced valence in the generating scenario. These negative estimates arose because the weights on $PE_{out,Q}$ and CC_{out} merely reflect the negated effects of $Q_{ch,out}$ and r_{unch}, respectively (Equation S20).

In contrast, the vector-based approach recovered the correct pattern. The median recovered length for the $\mathbf{R_{out}}$ vector was approximately 0.5, matching the true effect of reward. The median recovered lengths for the $\mathbf{PE_{out,Q}}$ and $\mathbf{CC_{out}}$ vectors were both 0, correctly indicating that neither prediction errors nor counterfactual comparisons contributed to valence in the generating scenario. The positive effects of $Q_{ch,out}$ and r_{unch}, which do not correspond to the predictions of any theory, were appropriately absorbed into the residual rather than distorting the vector length estimates. In summary, this simulation illustrates how the aggregated variable approach can yield highly misleading effect estimates, while the vector-based approach yields accurate and coherent results.

Figure S1

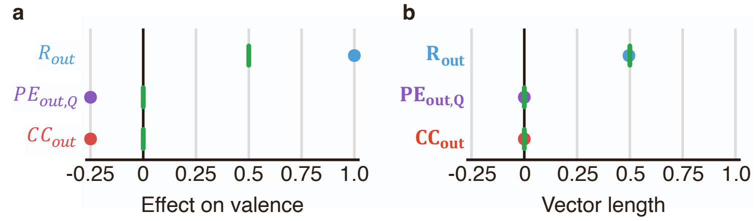

Estimates from vector-based and aggregated variable analyses, related to Figure 4. Plots display the results of applying two different analysis methods (aggregated variable and vector-based) to 200 simulated Study 1 datasets. **a.** Green tick marks show the (population mean) effect of each variable on affective valence in simulations. Colored dots show the effects estimated by the aggregated variable analysis. Specifically, these dots represent the median estimated values across the 200 simulated datasets (since the key result was the typical estimate generated by the method, we plot only medians for clarity and simplicity). The divergence between the actual and estimated effects indicates that the aggregated variable approach yielded inaccurate (or, at minimum, misleading) results. **b.** Green tick marks show the length of each vector in simulations. Although the simulation code did not set vector lengths explicitly, a length of 0.5 for $\mathbf{R_{out}}$ corresponds to an effect of 0.5 for the variable R_{out} = r_{ch} (see Tables 1, 2), and lengths of 0 for $\mathbf{PE_{out,Q}}$ and $\mathbf{CC_{out}}$ imply no effect of the corresponding variables. Colored dots show the lengths estimated by the vector-based approach (specifically, the median estimated value across the simulated datasets, as in panel A). The close correspondence between actual and estimated lengths indicates that the vector-based method yielded accurate results.

Data S3: Replicating results using alternatives to V_{block}

The prediction error theory implies that the affective impacts of choices should depend on the value of the chosen cue Q_{ch} relative to prior (pre-trial) expectations of reward. In Studies 1 and 2, we assumed these prior expectations were learned according to Equation 3, and termed them V_{block}. However, pre-trial reward expectations could be estimated in different ways, reflecting different assumptions about how they are formed.

To ensure that our findings regarding the determinants of valence were robust to different assumptions, we constructed four alternative models that each replaced V_{block} with a different estimate of pre-trial reward expectations: the mean reward from all past trials in the current block, the mean reward from all past trials in the task, a version of V_{block} that was updated using an independent learning rate

(Equation S18), and the average Q-value of all cues c in the current block, weighted according to the likelihood of the cue being chosen:

$$V_{\pi} = \frac{1}{n_{pairs}} \sum_{c} p(\text{choose } c) \cdot Q_c \quad (21)$$

where n_{pairs} is the number of cue pairs per block, and $p(\text{choose } c)$ is the probability of choosing cue c if its pair is presented (calculated according to Equation 8). We fit each of these models to data from Studies 1 and 2, and re-estimated the contributions of reward, prediction errors, and counterfactual comparisons to affective valence. In both studies, we replicated our finding that all three factors contribute to valence using all four alternative models, confirming that this finding is robust to different assumptions regarding pre-trial reward expectations (Figure S2).

Data S4: Considering the effect of cue pair presentations on valence ratings

In Study 1, we considered how the cues participants chose and the outcomes they received impacted their affect. We focused on these stimuli because past studies suggest they are the key determinants of valence in reward-based decision-making tasks like ours [S9-S10], and because the three theories of interest make clear and distinct predictions about how these stimuli will impact valence. However, it's possible that the presentation of cue pairs at the start of each trial also impacted participants' affect. To explore this possibility and its implications for our results, we re-analyzed the data from Study 1 on the assumption that cue pair presentations, in addition to choices and outcomes, influenced valence ratings.

If cue pair presentations impact affect, the reward theory would predict that pairs associated with greater expected reward would elicit more positive valence. The expected reward from a cue pair V_{pair} can be estimated as the expected reward of each cue in the pair, weighted by their likelihood of being chosen:

$$V_{pair,t} = p(\text{choose } a) \cdot Q_{a,t} + p(\text{choose } b) \cdot Q_{b,t} \quad (22)$$

where $p(\text{choose } a)$ and $p(\text{choose } b)$ are calculated according to Equation 8. The reward theory would predict that V_{pair}, in addition to Q_{ch} and r_{ch}, would influence valence ratings.

In contrast, the assumption that cue pair presentations impact affect would not change the predictions of the other two theories. The counterfactual comparison theory predicts no affective response to cue pair presentations, because participants' choices do not affect which pair is presented, and therefore there is no difference between the pair a participant receives and the pair they would've received had they made different choices. It could be argued that the prediction error theory implies an effect of pair presentations on valence, since cue pairs could elicit the prediction error $V_{pair} - V_{block}$. However, this would be immediately followed by the prediction error $Q_{ch} - V_{pair}$ upon choice, such that the overall (summed) prediction error elicited by viewing and choosing between cues would be $Q_{ch} - V_{block}$. Our original analyses already assume that the prediction error theory implies an effect of $Q_{ch} - V_{block}$ on valence (see PE_{ch} Table 1). Accordingly, to capture the potential effects of cue pair presentations on affect, we can simply add V_{pair} to our model of valence:

$$valence_t = \mathbf{w_k} \cdot \sum_{i=1}^{t} \gamma^{t-i} \mathbf{k}_i + \mathbf{w_z} \cdot \mathbf{z}_t + \epsilon_t$$
$$\mathbf{k_i} = \left[V_{block,i}, V_{pair,i}, Q_{ch,choice,i}, Q_{unch,i}, V_{trial,i}, Q_{ch,out,i}, r_{ch,i}, r_{unch,i}\right]^{\mathsf{T}} \quad (23)$$

We fit this model to the data from Study 1, finding that the addition of V_{pair} did not improve model fit ($LOOIC_{diff} = 1$, $SE_{diff} = 3.7$). This suggests that our original model – which assumes only choices and outcomes influence valence – provides a more parsimonious description of the data. Nonetheless, we sought to verify that our findings were robust to this assumption, so we re-estimated vector lengths based on the effects estimated in Equation S23:

$$\widehat{\mathbf{w}}_{\mathbf{k}} = m_1 \mathbf{R_{pres}} + m_2 \mathbf{R_{choice}} + m_3 \mathbf{PE_{choice}} + m_4 \mathbf{CC_{choice}} + m_5 \mathbf{R_{out}} + m_6 \mathbf{PE_{out,Q}} + m_7 \mathbf{PE_{out,V}} + m_8 \mathbf{CC_{out}} \quad (24)$$

where $\mathbf{R_{pres}}$ is a vector that predicts a positive effect of V_{pair} on valence and no effect of any other variable. As before, we calculated the ratio of each vector's length to the length of the RL variable effect vector $\mathbf{w_k}$, and summed vectors associated with the same theory so that each theory was represented by a single vector. We replicated our finding that the reward (median: .20[.07,.34], *pd* > 99.9%), prediction error (median: .47[.27,.58], *pd* > 99.9%), and counterfactual comparison (median: .26[.10,.43], *pd* >

99.9%) theories each explained unique portions of the RL variable effects. Moreover, we did not find evidence that $\mathbf{R}_{\mathbf{pres}}$ had positive length (median: .08 [0, .24], *pd* = 79.2%), confirming that our original analyses provide a parsimonious account of the data. Altogether, the results in this section validate our assumption that valence was driven by choices and outcomes, and verify that our key findings hold if different assumptions are made.

Figure S2

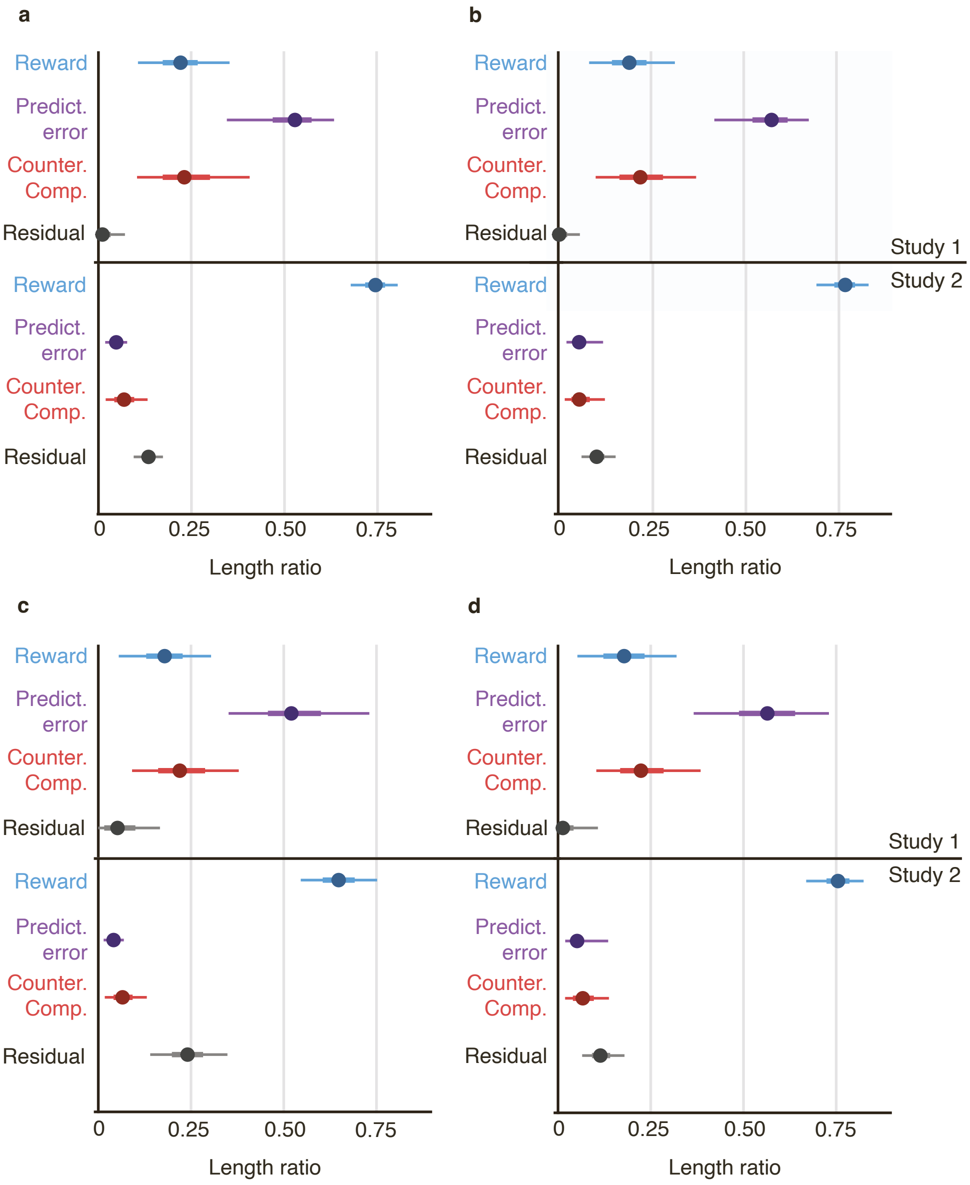

Theory vector lengths under models that use alternatives to V_{block}, related to Figure 4. Lengths were calculated in Manhattan distance, and then divided by the length of the RL variable effect vector, producing a "length ratio." Dots represent the posterior median of the estimate; thick lines represent 50% CIs; thin lines represent 95% CIs. In each panel, the estimates from Study 1 are in the top half of the plot and estimates from Study 2 are in the bottom half. **a**. Lengths of theory vectors when V_{block} was replaced with the mean reward from past trials in the block. **b.** Vector lengths when V_{block} was replaced with the mean reward from all past trials in task. **c.** Vector lengths when V_{block} was calculated according to Equation S18. **d.** Vector lengths when V_{block} was replaced with V_{π} (Equation S21).

Data S5: The effects of reward and valence on choice are robust to modeling assumptions

In this section, we verify that the effects of reward and affect associations on choice are robust to different modeling assumptions. First, we assessed whether these effects were dependent on the assumptions of our valence model by using a model-free operationalization of affective responses. Specifically, we fit models that updated affect associations using raw valence ratings (Equation S12), rather than using model-predicted affect (Equations 7, 10), to choices in Studies 2 and 3 (we considered only Studies 2 and 3 because participants did not explicitly report affective responses to specific outcomes in Study 1). For Study 2, we fit this model only to the block of trials in which participants rated their affective responses to outcomes. In both Study 2 and Study 3, reward and affect associations had positive effects on choice when estimated in this way, indicating that these results do not depend on the assumptions of our valence models (Study 2 β_A median: .29[.17,.42], *pd* > 99.9%; Study 2 β_Q median: 1.38[1.15,1.65], *pd* > 99.9%; Study 3 β_A median: .24[.18,.30], *pd* > 99.9%; Study 3 β_Q median: .57[.47,.67], *pd* > 99.9%).

Next, we tested whether these results were robust to assumptions of our choice models. Specifically, we assessed whether the effects of reward and affect associations on choice depended on the inclusion of the autocorrelation term S. This term captured a substantial amount of choice variability across all three studies, as evidenced by large improvements in predictive performance when S-values were added to the choice models (Table S1). Given the strong impact of this variable on choice predictions, we sought to verify that our key results held when it was removed from the models. For each study, we fit a model that was identical to the model used to estimate the effects of Q and A values in the main manuscript (Equations 8, 25), but with the choice autocorrelation term removed (i.e., in effect, β_S was fixed to 0 for all participants). In all three studies, we replicated our finding that both reward associations (Study 1 β_Q median: 1.16 [.84, 1.55], *pd* > 99.9%; Study 2 β_Q median: 2.18 [1.83, 2.58], *pd* > 99.9%; Study 3 β_Q median: .29 [.14, .44], *pd* > 99.9%) and affect associations (Study 1 β_A median: .21 [.03, .39], *pd* = 99.2%; Study 2 β_A median: .63 [0.38, 0.89], *pd* > 99.9%; Study 3 β_A median: 0.54 [.41, .67], *pd* > 99.9%) had positive effects on choice. We even replicated our findings that reward associations had a greater effect than affect associations in Studies 1 and 2 (Study 1 median difference: 0.95 [.56, 1.39], *pd* > 99.9%; Study 2 median difference: 1.55 [1.11, 2.03], *pd* > 99.9%), while affect associations had a greater effect than reward associations in Study 3 (median difference: 0.25 [-0.02, 0.51], *pd* = 96.7%). Thus, our key choice results did not depend on the inclusion of the autocorrelation term S.

Finally, to verify that our results did not depend on the assumptions of our RL models, we predicted choices using logistic regressions. On every trial in which a participant rated their affective response to a cue outcome, we used their valence rating and the reward value of the outcome to predict whether they would repeat their choice the next time the cue pair was presented (i.e., *stay*), as opposed to choosing the alternative cue (i.e., *switch*). We fit this model to Studies 2 and 3 only, since participants did not explicitly rate affective responses to specific outcomes in Study 1. Additionally, in Study 2, we included the counterfactual outcome as a third choice predictor (counterfactual outcomes weren't shown in Study 3). In both Studies 2 and 3, participants were more likely to repeat their choice when the reward was greater (Study 2 β_{rew} = .67, 95% CI [.58, .77], *p* < .001; Study 3 β_{rew} = .43, 95% CI [.35, .51], *p* < .001) and when the valence rating was more positive (Study 2 β_{val} = .30, 95% CI [.21, .38], *p* < .001; Study 3 β_{val} = .20, 95% CI [.15, .26], *p* < .001). Thus, our finding that reward and affect associations have positive influences on choice does not depend on the assumptions of our RL models.

Method S2: Vector length recovery analyses

To help validate our vector-based approach for inferring the determinants of affective valence, we conducted a parameter recovery analysis for vector lengths. Our objective was to determine whether our analytic method yields appropriate vector length estimates when applied to simulated task data where the ground truth is known. We simulated data based on our task and model, estimated vector lengths from the simulated datasets, and then compared the true and estimated lengths. Results indicated good recovery: estimated lengths clustered near their true values across a range of scenarios, both for vectors with a true length of 0 and for vectors with positive true length (Figure S3). These results support the validity of our method.

Simulations

We simulated responses to the Study 2 task in R [S11]. We based simulations on Study 2 rather than Study 1 because the Study 2 affect model (Equations 16 and 17) was simpler than the Study 1 affect model (Equation 5), which made large-scale recovery analyses more computationally tractable. We simulated 1,000 datasets, each with the same number of subjects and trials as in Study 2. In all simulations, the task structure was fixed to the Study 2 design: we used the same block and trial structure, and the same sequence of cue pair presentations, outcomes, and affect probes across trials.

Our simulations assumed that responses were generated according to the Study 2 model (see "Study 2: initial model" in Methods). For each simulated subject, parameter values were drawn from normal distributions whose means and SDs were set to the corresponding estimate from the fitted Study 2 model (specifically, to the posterior median of the estimate). For example, the mean of the β_Q distribution was set to its estimated population mean, and the SD of the distribution was set to its estimated population SD. As in the Study 2 model, we transformed constrained parameters after sampling (e.g., α was constrained to (0,1) by passing the value drawn from a normal distribution through a logistic function).

The only parameters not set in this way were the RL variable effects $\mathbf{w_x}$ (Equations 16, 17). To set $\mathbf{w_x}$, we first generated lengths for each of the 7 vectors representing specific predictions (the vectors defined in Table 2; e.g., $\mathbf{CC_{choice}}, \mathbf{R_{out}}$) and then translated those lengths into RL variable effects. We set vector lengths in four different ways, corresponding to four different conditions (250 datasets were simulated for each condition). In the "reward condition", the vector lengths for $\mathbf{R_{choice}}$ and $\mathbf{R_{out}}$ were set to be positive, and the remaining vector lengths were set to 0; this condition reflects a scenario in which affective valence is correctly described by the reward theory alone. Likewise, there was a "prediction error condition" in which the lengths for $\mathbf{PE_{choice}}$, $\mathbf{PE_{out,Q}}$, and $\mathbf{PE_{out,V}}$ were positive and the remaining lengths were zero, and a "counterfactual comparison condition" in which the lengths for $\mathbf{CC_{choice}}$ and $\mathbf{CC_{out}}$ were positive and the remaining lengths were zero. Finaly, we simulated data for a "blend condition", in which all vector lengths were set to be positive. This condition reflected a scenario in which the predictions from all theories explained equal portions of the RL variable effects. By simulating data under these four conditions, we could determine whether our vector-based analysis method yields valid results across a range of scenarios, and whether it is capable of detecting both the presence and absence of effects.

For each simulated subject, each nonzero vector length was sampled independently from a truncated normal distribution: *N*(0.5, 0.25) truncated to [0.05,0.95]. Notably, we sampled raw vector lengths, not "length ratios" (which are reported in the main manuscript) – length ratios are equal to the raw length of the vector divided by the length of the full vector of effects. We centered nonzero lengths at 0.5 because, in Study 2, affective valence was primarily determined by reward, and the vector lengths for $\mathbf{R_{choice}}$ and $\mathbf{R_{out}}$ were approximately 0.5 in this study. We used a relatively large SD for vector lengths – equal to half the mean length – to allow substantial between-subject heterogeneity. However, we truncated the distribution from below at 0.05 to ensure strictly positive lengths, in keeping with the intended generating scenario (e.g., according to the reward theory, vector lengths for $\mathbf{R_{out}}$ and $\mathbf{R_{choice}}$ should be positive, so we restricted these lengths to be positive in the reward condition). We truncated distributions from above at 0.95 to maintain symmetry.

For each subject, we used the sampled vector lengths to determine the RL variable effects. Specifically, we scaled each direction vector (Table 2) by its sampled length, and then summed these scaled vectors to derive the overall effect of each RL variable. The resulting RL variable effects $\mathbf{w_x}$ were used to generate valence ratings according to Equations 16 and 17.

Recovery analyses

For each simulated dataset, we estimated the RL variable effects from the simulated valence ratings, and then estimated the length of each vector following the method described in the main manuscript. To keep these analyses computationally tractable across 1,000 datasets, we did not refit the full hierarchical Bayesian model used for Study 2. Instead, we fit frequentist linear models with subject fixed effects, using the simulated RL variables as observed inputs.

More concretely, we fit two regressions per dataset which matched the structure of Equations 16 and 17: one regression predicted post-choice valence ratings and the other predicted post-outcome ratings. The post-choice model included V_{block}, $Q_{ch,choice}$, and Q_{unch}, plus the same nuisance terms used in the Study 2 valence model (block number, trial number, and previous rating) and subject fixed effects.

The post-outcome model included V_{trial}, $Q_{ch,out}$, r_{ch}, and r_{unch}, with the same nuisance terms and subject fixed effects. This yielded recovered point estimates for the seven RL variable effects.

This estimation strategy diverged from our primary Study 2 analysis (hierarchical Bayesian estimation) for practical reasons. Fitting 1,000 hierarchical Stan models was not feasible for this validation exercise. Therefore, we treated the simulated RL variables as known so that their effects could be estimated through frequentist regression. Because our objective here was to validate the vector-based analysis method, rather than to validate the RL model or to conduct a power analysis, we considered this an acceptable simplification.

We used subject fixed effects rather than mixed-effects models to ensure stable, fully automated estimation across all simulated datasets. Mixed-effects fits were expected to produce occasional convergence or singularity issues that would complicate a large-scale simulation workflow. Subject fixed effects provided a robust alternative that still controlled subject-specific baseline differences.

After estimating the seven RL variable effects, we used the L-BFGS-B optimization algorithm [S12], implemented in R's 'optim' function [S11], to identify the set of non-negative vector lengths that produced the best approximation to the estimated effects (Equation 6); this mirrored the analysis method used in the main manuscript. For each simulated dataset, we conducted vector optimization using point estimates of the RL variable effects obtained from the recovery regression, yielding point estimates of vector lengths.

Results

Recovered vector lengths are shown in Figure S3. As can be seen in this figure, recovered lengths clustered near their true values in all four conditions, both for vectors with true length 0 and for vectors with positive true length (note the white diamonds, which represent median recovered lengths). Any apparent bias was small, and generally conservative: positive vector lengths were more often underestimated (56.4% of recovered lengths) than overestimated (43.6%), and the most commonly overestimated quantity was the unexplained residual (86.5%). Further, zero lengths were frequently estimated with precision: for true lengths of 0, the majority of recovered lengths – 57.8% – were exactly 0 (not merely small values). Likewise, in 11 out of 14 cases, the median recovered length for vectors with a true length of 0 was precisely 0. Overall, these results support the validity of our vector-based analysis method for identifying the determinants of affective valence (under the assumptions of our simulation framework)

Because our method yields accurate estimates of vector lengths, it also allows for accurate comparisons between vector lengths to assess the relative explanatory power of different predictions and theories. In the three conditions where data were generated according to a single theory's predictions (panels a, b, and c of Figure S3), all vectors associated with the generating theory had greater length than all vectors associated with the alternative theories in 100% of simulations. Additionally, for these three conditions, we summed fitted vectors within each theory and compared the lengths of the resulting theory-level vectors to directly test which theory had the greatest explanatory power (as we did in the main manuscript: see "Study 1 results" and "Comparing vector lengths across studies"). In 100% of simulations, the estimated vector length for the generating theory was greater than the estimated lengths for the alternative theories – mirroring the results of the prediction-level vector length comparisons. Thus, our analysis method consistently correctly identified the predictions and theories that best explained the results.

Figure S3

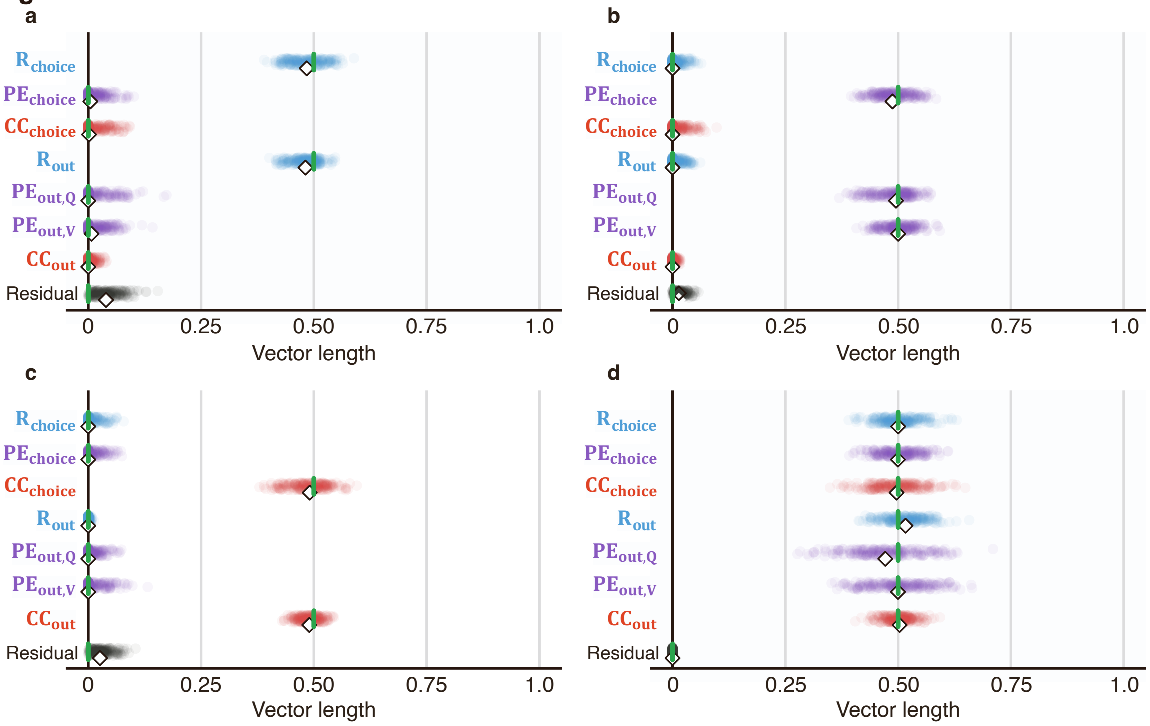

Recovered vector lengths, related to Figure 4. Estimates of vector lengths (raw lengths, not length ratios) from four different simulation conditions. Green tick marks represent the true length of each vector within each simulation condition. Each semi-transparent colored dot represents the estimated length of the corresponding vector for one simulated dataset (there were 250 simulations per condition). White diamonds represent the median estimates for each vector length within each condition. **a.** The "reward condition," in which data were simulated according to the predictions of the reward theory. **b.** The "prediction error condition," in which data were simulated according to the predictions of the prediction error theory. **c.** The "counterfactual comparison condition," in which data were simulated according to the predictions of the counterfactual comparison theory. **d**. The "blend condition," in which data were simulated according to the predictions of all theories. The close correspondence between actual and recovered lengths across all conditions supports the validity of the vector-based analysis method used here.

Method S3: Vector length estimation validation

To verify that our optimization algorithm correctly recovers vector lengths from a given draw of RL variable effects $\mathbf{w_x}$, we constructed five hypothetical draws in which the true vector lengths were known, and checked whether the L-BFGS-B algorithm recovered the correct lengths. Each hypothetical draw was constructed by scaling each direction vector (Table 2) by an assigned length and summing the results (Equation 6), yielding a $\mathbf{w_x}$ vector with known generating lengths. Each hypothetical draw represented a different scenario. In the first three scenarios – "reward only," "prediction error only," and "counterfactual comparison only" – only the vectors associated with the corresponding theory were assigned positive length (specifically, a length of 0.5), while all other vectors were assigned length 0. In the fourth scenario ("all positive"), all vectors were assigned positive length (0.5). These four scenarios match the four conditions used in the vector length recovery analyses reported above in "Vector length recovery analyses." In the fifth scenario ("all reversed"), we multiplied each vector by a negative value (-0.5) to produce a pattern of RL variable effects that contradicted every vector's predictions. Because no vector's predictions were consistent with these effects, the correct length for every vector was 0 in this scenario.

To assess whether the optimizer recovered the correct lengths, we compared the estimated lengths to the true lengths implied by each hypothetical draw. For positive true lengths, we required that the estimated length be correct within a tolerance of 10^{-4} (i.e., to four decimal places, which is two decimal

places beyond the precision reported in the manuscript); this tolerance was necessary because the optimizer was not perfectly exact for positive values. For true lengths of 0, we required that the estimated length be exactly 0 – not merely a near-zero value. The L-BFGS-B algorithm is designed to satisfy this requirement: it first identifies any variables whose optimal values lie on the boundary (here, vector lengths that should be exactly 0) before optimizing the remaining variables [S12]. Additionally, in the manuscript, we report the posterior probability that a vector's length is positive; valid estimation of this probability requires that lengths of 0 are recovered with precision.

In all five scenarios, the optimizer recovered the correct length for every vector: positive lengths were recovered within the specified tolerance, and zero lengths were recovered exactly. This confirms that our length estimation procedure correctly infers the portion of RL variable effects captured by each vector across a range of situations (i.e., a range of potential posterior draws).

To illustrate how correct inference on individual posterior draws yields valid posterior distributions, we analyzed a simplified scenario in which the correct posterior distribution for vector length was self-evident. For this scenario, we assumed that r_{ch} (the reward from the chosen cue) was the only variable that influenced valence, and we estimated the length of only one vector: $\mathbf{R_{out}}$ (which represents the reward theory's prediction that r_{ch} will have a positive effect). We constructed a hypothetical posterior distribution for the effect of r_{ch} by drawing 10,000 samples from a normal distribution centered at 0 (SD = 0.1; Figure S4a). Because $\boldsymbol{R_{out}}$ predicts a positive effect of r_{ch}, the estimated posterior probability that $\boldsymbol{R_{out}}$ has positive length should (approximately) equal the posterior probability that the effect of r_{ch} is positive; in the posterior we constructed, the latter probability was 49.3% (i.e., 49.3% of simulated r_{ch} draws were positive). We estimated the length of $\boldsymbol{R_{out}}$ from these posterior draws, and found that its estimated probability of being positive closely matched the true probability: 49.2%. The slight underestimation (49.2% estimated vs. 49.3% true) occurred because the optimizer is not exact for positive values, and thus occasionally returned a length of 0 for very small positive r_{ch} draws. Otherwise, as Figure S4 shows, positive draws for the effect of r_{ch} yielded positive length estimates, while non-positive draws yielded length estimates of exactly 0 – resulting in an accurate estimate for the posterior probability of positive length. This example demonstrates how our optimization procedure translates a valid posterior distribution over RL variable effects into a valid posterior distribution over vector lengths.

Figure S4

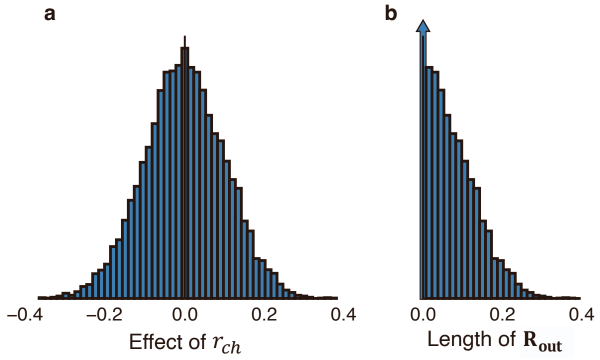

Example posterior distributions for r_{ch} and $\mathbf{R_{out}}$, related to STAR Methods. Plots illustrating how posterior distributions for RL variable effects translate into posterior distributions for vector lengths. These plots are based on a simplified, hypothetical scenario in which only r_{ch} influenced affective valence, and in which only $\mathbf{R_{out}}$ was used to explain this effect. **a.** A hypothetical posterior distribution for the effect of r_{ch} on valence. The plot displays a histogram of 10,000 posterior draws for this effect, which were sampled from the distribution Normal(0,0.1). **b.** A posterior distribution for the length of $\mathbf{R_{out}}$, constructed from the posterior distribution shown in panel A. For each posterior draw of the effect of r_{ch}, the L-BFGS-B optimization algorithm was used to identify the (non-negative) length of $\mathbf{R_{out}}$ that provided the optimal approximation to the sampled r_{ch} effect. Note the upward arrow for the column centered on 0; 49.2% of $\mathbf{R_{out}}$ lengths were exactly 0, resulting in a spike at this value that did not fit in the plot. As these plots illustrate, non-positive draws of r_{ch} resulted in $\mathbf{R_{out}}$ length estimates of 0, while positive draws resulted in length estimates that matched the corresponding r_{ch} effect estimate (such that the positive half of the two distributions are nearly identical).

Data S6: Verifying the effect of reward associations on choice

In the models reported in the main article, we update affect associations *A* using estimates of the affective impacts of cue outcomes. Because these estimates are noisy and imperfect, it's possible that we found a positive effect of reward associations on choice only because reward predicts valence, and thus can serve as a second estimate of the affective impacts of outcomes. In other words, it's conceivable that only affect associations influence choice, and the apparent effect of reward associations is spurious, merely reflecting the ability of reward to predict affect. To investigate this possibility, we compared the effects of reward associations observed in our studies to the effects of reward associations observed in simulations that assumed only affect associations influence choice. In all studies, the effect of reward associations was substantially greater in the actual data than in the simulated data (Figure S5a), implying that reward associations have an independent, positive effect on choice.

Simulations and analyses

For the simulations, we fit models to Studies 1, 2, and 3 that assumed only affect associations *A* influenced choice, with no effect of reward associations. These models were identical to those reported in the main article (Equations 8, 25), except the effect of reward associations β_Q was fixed to 0 for all participants. We simulated 4,000 datasets from the posterior distribution of each model.

For each dataset, we estimated the effect of reward associations on choice using an analysis method that mimicked the models reported in the main article, but which was more computationally efficient. This method used frequentist regression to estimate effects, rather than using hierarchical Bayesian parameter estimation. We began by estimating reward associations Q according to Equation 1, setting the learning rate α to the value estimated in the model from which the data was simulated (to avoid having to re-estimate learning rates for each dataset). We then modeled valence according to Equations 5, 9, and 17, but we fit these models by simply regressing valence ratings onto valence predictors, fitting a separate regression to each participant's data. For the Study 1 model (Equation 5), we assumed that the decay rates γ matched the values estimated in the model from which the data was simulated. These valence regression models provided estimates of the effects of RL variables on valence $\mathbf{w_x}$, which we used to estimate the affective impacts of cue outcomes. These estimated affective impacts were used to update affect associations A (Equations 7, 10). Finally, we modeled choice according to Equations 8 and 25, but we fit these models by simply regressing choices onto choice predictors, collapsing all participants' data into a single regression model.

This analysis method yielded an estimated effect of reward associations on choice for each simulated dataset. We also applied this approximate analysis method to the real data from each study, estimating the actual effects of reward associations on choice.

Results

In all three studies, the actual effect of reward associations was substantially greater than the effects estimated from simulated data (Figure S5a). This verifies that reward associations have positive effects on choice, over and above what can be explained by the relationship between reward and affect.

There is one, potential alternative explanation for these results: if the model from which we simulated data underestimated the true effect of affect associations on choice, this could've caused us to find smaller effects of reward associations in the simulated data than in the real data. If this were the case, we'd also expect to find smaller effects of affect associations in the simulated data than in the real data. However, in all three studies, we found *larger* effects of affect associations in the simulated data, ruling out this possibility (Figure S5b). Indeed, this result suggests that the model from which we simulated data *overestimated* the effect of affect associations on choice, implying that our simulations may exaggerate how large the estimated effect of reward associations could be if reward associations did not truly influence decision-making. Altogether, our analyses demonstrate that the effects of reward associations observed in all three studies are considerably larger than what would be expected if only affect associations influenced choices.

Figure S5

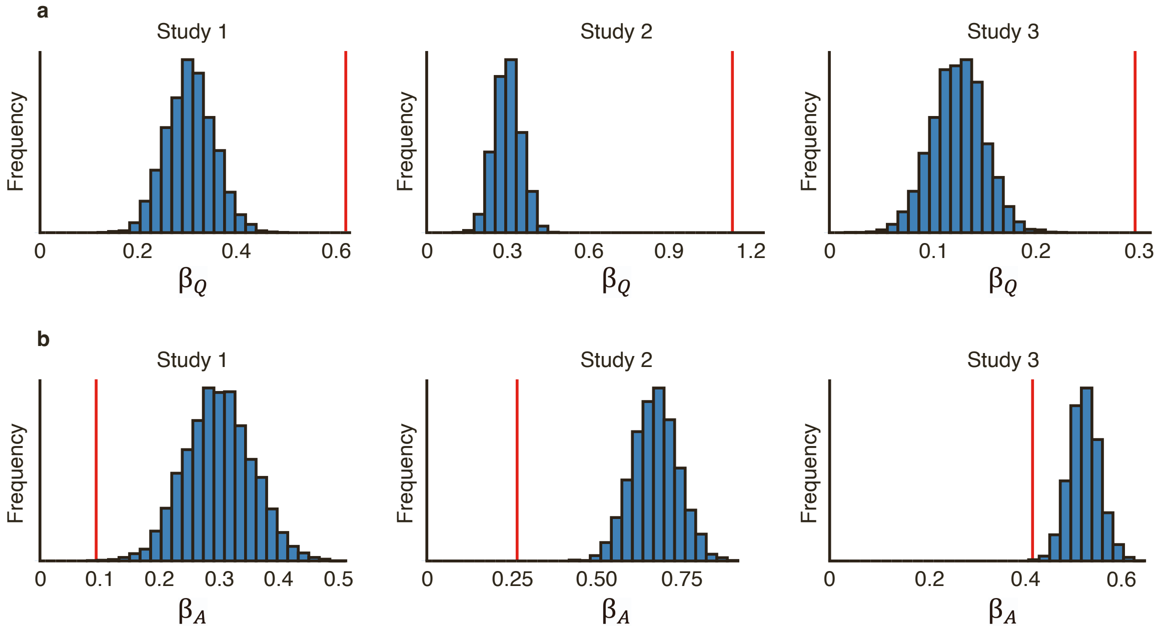

Simulated effects of choice predictors assuming no influence of reward associations. We fit a model to the data from each study which assumed that affect associations - but not reward associations - influenced choice, and simulated 4,000 datasets from each model. We then estimated the effects of reward and affect associations on choice in each of the simulated datasets. **a**. Histograms showing the estimated effects of reward associations on choice in the simulated datasets. The red lines show the estimated effects of reward associations in the actual data. In all three studies, the actual effect of reward associations was greater than all 4,000 simulated effects, indicating that the actual effects are larger than what would be expected if only affect associations influenced choice. **b.** Histograms showing the estimated effects of affect associations on choice in the simulated datasets. In all three studies, the actual effect of affect associations (red lines) was smaller than at least 99.9% of the simulated effects. This confirms that the model from which we simulated data did not underestimate the effect of affect associations on choice.

Data S7: Comparing predictive performance of models with and without A

In the main article, we asked whether affect reinforced choice alongside external reward. To answer this question, we modeled affect associations A and tested whether they had a positive effect on choice likelihood (while controlling for reward associations Q). Here, we consider a different, but related, question: does incorporating affect associations into RL models improve their ability to predict choices in tasks like ours? To address this question, we compared the out-of-sample predictive accuracies of models with and without affect associations using PSIS-LOO-CV [S1] – a procedure which efficiently estimates the leave-one-out cross-validation performance of a model.

For each study, we compared a model that included an effect of A (the models described under *"Studies 1 and 2: adding affect associations"* and *"Study 3 model"* in Methods) to an otherwise identical model that excluded this effect, evaluating each model's ability to predict held-out choices (LOOIC values were computed from choice predictions only). In all three studies, the model which included an effect of affect associations had better estimated out-of-sample predictive performance than the model which did not, as indicated by lower LOOIC values (Table S3). In Studies 2 and 3, the improvement in predictive performance was several times the standard error of the comparison, indicating high confidence that including A values improved the models' predictions. In Study 1, the improvement was smaller and only 1.3 times the standard error of the difference. Thus, although Study 1 was directionally consistent with the other studies, it provides weaker evidence that including affect associations improves out-of-sample choice prediction. Several features of Study 1 may have limited the predictive benefit of including A, including relatively noisy affect ratings (R^2 for post-outcome ratings was .44 in Study 1, compared to .86 in Study 2 and .78 in Study 3), the strong overlap between Q and A values (discussed in the Results,

"Investigating the role of affect in RL"), and relatively limited variance in A values (also discussed in "Investigating the role of affect in RL").

Taken together, these comparisons indicate that incorporating affect associations into Q-learning models tends to improve out-of-sample choice predictions in paradigms like ours. Simultaneously, they show that the magnitude and reliability of this improvement varies across task and model designs.

Table S3

a

Model	$LOOIC_{diff}$	SE_{diff}	$LOOIC_{diff}$ / SE_{diff}
With A	0		
No A	8.0	6.3	1.3

b

Model	$LOOIC_{diff}$	SE_{diff}	$LOOIC_{diff}$ / SE_{diff}
With A	0		
No A	76.7	17.5	4.4

c

Model	$LOOIC_{diff}$	SE_{diff}	$LOOIC_{diff}$ / SE_{diff}
With A	0		
No A	111.6	24.1	4.6

Comparison of models with and without A, related to Figure 10. Comparisons of models that did and did not allow for an effect of affect associations A on choice. Panel A shows results for models fit to Study 1, panel B for models fit to Study 2, and panel C for models fit to Study 3. $LOOIC_{diff}$ is the difference between the LOOIC of the current model and the model with A. These values indicate that, in all studies, including affect associations A in the model improved its expected out-of-sample predictive performance (for choices). SE_{diff} is the standard error of $LOOIC_{diff}$, and $LOOIC_{diff}$ / SE_{diff} is the ratio of the two.

<u>**Data S8: Comparing learning and task engagement across studies**</u>

Cue outcomes were smaller and somewhat noisier in Study 3 than in Studies 1 and 2, which conceivably could have reduced task engagement or participants' motivation to learn. To address this concern, we compared measures of attention and learning across the three studies. The results indicate that learning and task engagement were as strong in Study 3 as in Studies 1 and 2 – and were in some respects stronger.

Attention measures

To assess participants' overall level of task engagement, we compared attention metrics across the three studies. For these comparisons, we considered all participants, not merely those who passed data quality criteria, since attention metrics were among the quality criteria used.

As our primary measure of attention, we considered the percentage of attention checks completed successfully in each study. Over 95.5% of attention checks were passed in Study 3, while 88.1% of checks were passed in Study 1 and 97.3% of checks were passed in Study 2. Thus, participants passed checks at a high rate in Study 3, comparable to the rates observed in the other studies.

As secondary measures of attention, we also considered the percentage of choices and affect ratings made within the allotted time; a higher percentage of choices and ratings completed suggests a higher level of task engagement. We found that participants completed a very high percentage of both choices and ratings in Study 3, similar to the percentages in Studies 1 and 2. Specifically, Study 3 participants completed 98.9% of affect ratings – compared to 98.8% in Study 1 and 99.1% in Study 2 – and completed 97.2% of choices – compared to 95.7% in Study 1 and 96.3% in Study 2. Once again, these results suggest good attention among Study 3 participants, comparable to the attentiveness of Study 1 and 2 participants.

Learning

Next, we compared learning across the three studies. More specifically, we compared how strongly cue outcomes shaped subsequent choices and preferences. To make these comparisons, we examined parameter estimates from the primary model for each study – that is, from the model reported in the main article that included an effect of affect associations A on choice, where A was estimated via Equation 7 (in Studies 1 and 2) or Equation 10 (in Study 3). We compared learning in two different ways.

First, we compared the estimated learning rates α across studies. A relatively high learning rate means that each outcome had a relatively large effect on subsequent cue valuations (i.e., cue preferences). If participants made little effort to learn from outcomes, we would expect a very low learning rate, indicating that outcomes had little impact on subsequent preferences. However, we found a robust learning rate of .38 (95% CI [.20, .62]) in Study 3. This was higher than the estimated learning rates in Study 1 (median difference: .27[.08,.50], *pd* > 99.9%) and Study 2 (median difference: .26[.08,.50], *pd* = 99.9%). This result suggests that learning was at least as responsive to recent cue outcomes in Study 3 as in the other studies.

Second, we compared how likely participants were to choose cues that had previously led to greater reward and more positive affect across studies. One each trial t, the primary models estimated the effects of reward associations and affect associations on the likelihood of choosing cue a over cue b (Equations 8, 25); these effects were represented by the terms $\beta_Q(Q_{a,t} - Q_{b,t})$ and $\beta_A(A_{a,t} - A_{b,t})$, respectively. For each study, we quantified the average, combined influence of these effects on choice likelihood across trials as:

$$s = \frac{1}{T}\sum_{t=1}^{T}\left(\beta_Q(Q_{a,t} - Q_{b,t}) + \beta_A(A_{a,t} - A_{b,t})\right)^2 \quad (25)$$

where T is the total number of trials in the study. A higher value of s indicates that participants had a stronger tendency to select cues which previously led to greater reward and more positive affect in that study.

If participants learned less from task experience in Study 3 than in Studies 1 and 2, we'd expect a relatively low value of s in Study 3, suggesting that participants did relatively little to adapt their behavior in response to past outcomes (i.e., in response to past reward and affect). Instead, we found that the value of s in Study 3 fell between the values of s in the other studies: s was marginally higher in Study 3 than in Study 1 (median difference: .07 [-.17,.29], *pd* = 71.8%), but smaller in Study 3 than in Study 2 (median difference: -3.36 [-4.32,-2.67], *pd* = 99.9%). These results suggest that choices were *not* particularly insensitive to past outcomes in Study 3. Instead, they suggest that learning in Study 3 was comparable to learning in the other studies.

Method S4: Choice effect parameter recovery

Given the large number of choice predictors in the winning Study 3 model (Equation 25), and the overlaps between these predictors (e.g., the shared variance between reward and affect associations), we conducted a parameter recovery analysis to verify that the effects of interest were identifiable. Specifically, we assessed whether our analysis method could recover low-bias estimates of the effects of reward associations (β_Q), affect associations (β_A), and residual associations (β_R) on choice from simulated data generated under a range of effect sizes. The results indicated good recovery (and thus strong identifiability): estimated effects of β_Q, β_A, and β_R clustered closely around their true values (Figure S6).

Simulations

We simulated 250 datasets using the Study 3 task structure (i.e., using the same number of subjects and trials, the same trial structure, and the same sequence of cue pairs, box amounts, and outcomes that appeared in the real data). Data were generated from the Study 3 model (see "Study 3 model" in Methods). For each simulated dataset, subject-level parameters were drawn from normal, population-level distributions; for each parameter, the mean and SD of the distribution were set to the posterior medians of the parameter's population mean and SD estimated from the Study 3 data. As in the fitted Study 3 model, constrained parameters (e.g., learning and decay rates) were transformed after sampling by passing the sample through a logistic function.

The only parameters whose population means were not anchored to their fitted values were the three focal choice effects: β_Q, β_A, and β_R. For each simulated dataset, the population mean of each focal

effect was drawn independently from a uniform distribution spanning $[-1.5|\hat{\theta}|, 1.5|\hat{\theta}|]$, where $\hat{\theta}$ is the posterior median of the population mean estimated from the data. This range ensured that we simulated both positive and negative effects of a similar size to the effects observed in the data.

On each trial of each simulation, choices were generated according to Equation 25, and affect was generated according to Equations 9 and 20 (as in the Study 3 model). Residual noise was drawn from a normal distribution whose SD was set to the posterior median of the estimated residual SD. After each trial, all association values (e.g., reward associations Q, affect associations A) were updated according to the same learning and decay rules used in the fitted model.

Recovery analyses

For each simulated dataset, we estimated the effects of reward, affect, and residual associations on choice using logistic regression (which is equivalent to the softmax choice rule used in the Study 3 model) with subject fixed effects. The regression predicted choices using the same eight predictors shown in Equation 25 (with side bias represented by the intercept). We used subject fixed effects, rather than hierarchical Bayesian estimation or mixed-effects models, to ensure computational tractability and stable, fully automated estimation across all simulated datasets (as in the vector length recovery analyses reported above).

For computational efficiency, the recovery regression used the true (simulated) choice predictors (e.g., the true Q and A values used to simulate choices), rather than re-estimating these quantities from the data (which would require fitting a computational model to each simulated dataset). Because our objective was to assess whether the three focal choice effects could be identified in the presence of several competing choice predictors – rather than to evaluate the RL or valence models, or to conduct a power analysis – we considered this an acceptable simplification.

We plotted the recovered logistic regression coefficients against the true values of the focal effects (β_A, β_Q, and β_R; Figure S6). Recovery was strong for all three effects: recovered estimates (dots) clustered closely around the true population means (45-degree line) for β_A, β_Q, and β_R. The only apparent bias was a modest attenuation of large effects for β_A (and possibly for β_Q and β_R, but the potential attenuation was less clear for these parameters). However, this bias was small and conservative (i.e., it would make large effects harder, not easier, to detect). Thus, this attenuation does not call into question our key affect-related choice findings – that affect associations had a positive effect on choice, and that their effect was greater than that of reward associations. Overall, these results support the validity of our results, indicating that the effects of reward, affect, and residual associations on choice are identifiable in the Study 3 model.

Figure S6

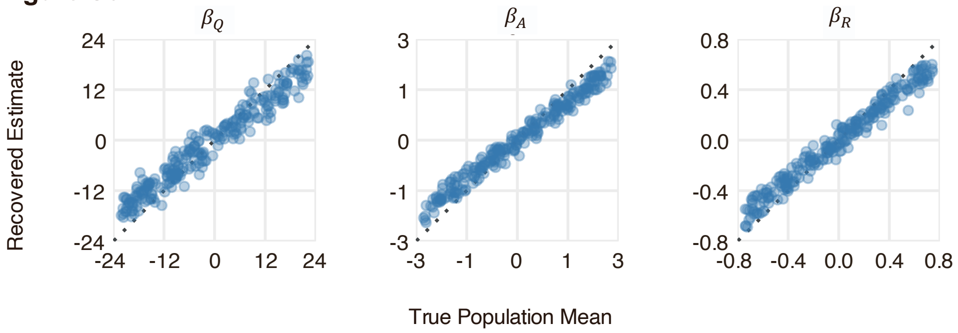

Parameter recovery for Study 3 choice effects, related to STAR Methods. Each panel shows the relationship between the true population mean (x-axis) and the recovered estimate (y-axis) for one of three choice effects: β_Q (effect of reward associations), β_A (effect of affect associations), or β_R (effect of residual associations). Dots represent individual simulations (*N* = 250); the dotted 45-degree line represents perfect recovery. All effects are unstandardized; thus, their scale differs from the effects reported in the main manuscript, which were standardized. For each effect, the true (simulated) population mean was drawn uniformly from a range spanning ±1.5 times the effect estimated from the

actual Study 3 data. The close proximity of recovered estimates to the 45-degree lines indicates good recovery for all effects.

Data S9: Divergences from preregistration

Study 2

We did not preregister the use of vectors to test the predictions of the reward, prediction error, and counterfactual comparison theories. We consider the vector-based analysis method to be conceptually and statistically superior to the preregistered approach. Nonetheless, the results of the preregistered analyses were similar to the results reported in the main article.

In our preregistered analyses, we regressed valence ratings onto the variables predicted to influence valence by the reward, prediction error, and counterfactual comparison theories. When participants rated their response to a cue, ratings were predicted by:

$$valence_t = \mathbf{w}_{\mathbf{x},\mathbf{choice}} \cdot \mathbf{x}_{\mathbf{choice},t} + \mathbf{w}_{\mathbf{z}} \cdot \mathbf{z}_t + \epsilon_t$$
$$\mathbf{x}_{\mathbf{choice},t} = \left[R_{choice,t}, PE_{choice,t}, CC_{choice,t}\right]^\top \quad (26)$$

When participants rated their response to an outcome, ratings were predicted by:

$$valence_t = \mathbf{w}_{\mathbf{x},\mathbf{out}} \cdot \mathbf{x}_{\mathbf{out},t} + \mathbf{w}_{\mathbf{z}} \cdot \mathbf{z}_t + \epsilon_t \quad (27)$$
$$\mathbf{x}_{\mathbf{out},t} = \left[R_{out,t}, PE_{out,Q,t}, PE_{out,V,t}, CC_{out,t}\right]^\top$$

We calculated trial-level values for the variables in $\mathbf{x}$ using the equations shown in Table 1. For example, the value of CC_{choice} on trial t was given by:

$$CC_{choice,t} = r_{ch,t} - r_{unch,t} \quad (28)$$

We found positive effects of R_{choice} (median: 0.66[.50,.89], *pd* > 99.9%), R_{out} (median: .64[.57,.72], *pd* > 99.9%), $PE_{out,V}$ (median: .03[.01,.06],], *pd* = 99.7%), and CC_{out} (median: .02[.01,.03], *pd* = 98.6%) on valence (Figure S7). This mirrors the results we report in the main article, in which each of these vectors had positive length.

Figure S7

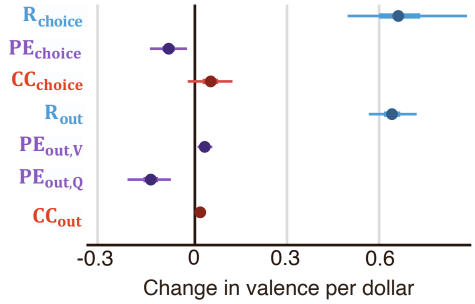

Effects of preregistered valence predictors, related to Figure 4. Effects of variables associated with the reward, prediction error, and counterfactual comparison theories on z-scored valence ratings. Dots represent the posterior median of the estimate; thick lines represent 50% CIs; thin lines represent 95% CIs. Each estimate reflects the change in valence induced by a $1 increase in the predictor variable.

In our preregistered analyses, we also tested the hypothesis that valence was best explained by a combination of the reward, prediction error, and counterfactual comparison theories. To do this, we fit a model for each theory that predicted valence ratings using only the predictions made by that theory. For example, the model for the reward theory predicted valence ratings as follows:

$$valence_{choice,t} = w_{R,choice} \cdot R_{choice,t} + \mathbf{w}_{\mathbf{z}} \cdot \mathbf{z}_t + \epsilon_t \quad (29)$$
$$valence_{out,t} = w_{R,out} \cdot R_{out,t} + \mathbf{w}_{\mathbf{z}} \cdot \mathbf{z}_t + \epsilon_t \quad (30)$$

where $valence_{choice}$ and $valence_{out}$ refer to post-choice and post-outcome valence ratings, respectively.

We compared these models to the model that combined the predictions of all theories (Equations 16, 17), and a null model that only included nuisance predictors. The reward, prediction error, and counterfactual comparison models fit better than the null, indicating that each theory captured a portion of valence. However, the combined model fit best, implying that valence is best described by a combination

of all three theories (Table S4). Thus, the model comparisons results are also consistent with the findings reported in the main article.

Table S4

Model	$LOOIC_{diff}$	SE_{diff}	$LOOIC_{diff}$ / SE_{diff}
Combined	0		
Reward	1064.6	86.7	12.3
Prediction error	1819.8	104.0	17.5
Counter. comparison	8702.5	220.4	39.5
Null	12798.7	259.9	49.3

Preregistered model comparisons, related to Figure . We constructed models based on the predictions of the reward, prediction error, and counterfactual comparison theories, and fit them to valence ratings from Study 2. We also fit a model that combined predictions from all three theories, and a null model that only included nuisance predictors. The combined model fit best. $LOOIC_{diff}$ is the difference between the LOOIC of the current model and the combined model. SE_{diff} is the standard error of this difference, and $LOOIC_{diff}$ / SE_{diff} is the ratio of the two.

Study 3

Our preregistered models used raw valence ratings to update affect associations (e.g., Equation S12). However, in the main article, we report results from a model that used model-predicted valence to update affect associations A (Equation 10), and that used valence rating residuals to update separate "residual associations" R (Equation 11). We found positive effects of reward and affect associations on choice using both models (see "The effects of reward and valence on choice are robust to modeling assumptions", above). Nonetheless, we prefer a model with separate effects of model-predicted and residual valence for two reasons.

First, the effect of model-predicted valence on choice β_A differed markedly from the effect of residual valence β_R (median difference: .40[.27,.53], *pd* > 99.9%), suggesting that these effects should be considered separately, rather than being represented by a single estimate based on gross valence ratings. Second, we had to use model-predicted valence to update affect associations A in Studies 1 and 2, because these studies did not include valence ratings after every outcome. Thus, by also using model-predicted valence to update affect associations in Study 3, we ensured that A values had a consistent interpretation across studies.

<u>**Additional figures and tables**</u>

Figure S8

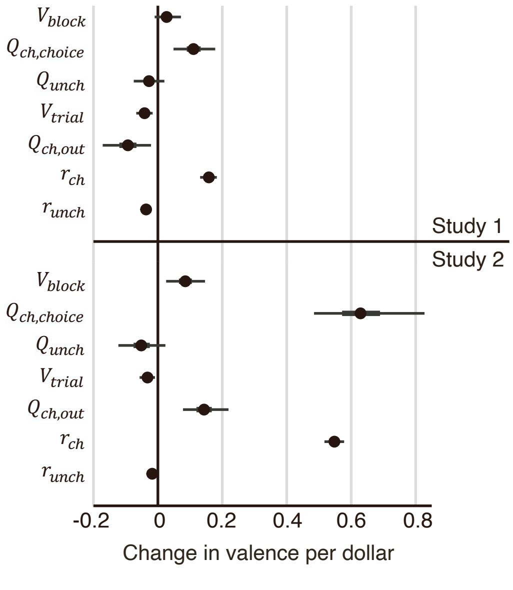

Effects of RL variables on valence, related to Figure 3. Estimated effects of RL variables on z-scored valence ratings. Dots represent the posterior median of the estimate; thick lines represent 50% CIs; thin lines represent 95% CIs. The estimates from Study 1 are in the top half of the plot and estimates from Study 2 are in the bottom half. Each estimate reflects the change in valence induced by a $1 increase in the RL variable. For example, the median effect of r_{ch} in Study 1 was .18, suggesting that a valence rating made immediately after receiving a $1 reward would be .18 standard deviations greater than a rating made after receiving $0.

Table S5

a

Theory vectors	Posterior probability
Reward, prediction error, counterfactual comparison	99.8%
Prediction error, counterfactual comparison	0.2%
Reward, prediction error	0.1%
Reward, counterfactual comparison	0.0%
Reward	0.0%
Prediction error	0.0%
Counterfactual comparison	0.0%

b

Theory vectors	Posterior probability
Reward, prediction error, counterfactual comparison	99.6%
Prediction error, counterfactual comparison	0.0%
Reward, prediction error	0.1%
Reward, counterfactual comparison	0.3%
Reward	0.0%
Prediction error	0.0%
Counterfactual comparison	0.0%

Posterior probabilities of theory combinations, related to Figure 4. For each combination of theory-level vectors, the probability that those – and only those – vectors had positive length (rounded to the nearest tenth). The set of vectors with positive length represents the combination of theories that best explains the RL variable effects. Results for Study 1 are in Panel A; results for Study 2 are in Panel B.

Table S6

a

Parameter	P_{50}	P_5	P_{95}
β_S	1.33	1.17	1.49
$\mathbf{w_z}[base]$	-0.04	-0.07	-0.02
$\mathbf{w_z}[type]$	0.01	-0.01	0.03
$\mathbf{w_z}[block]$	-0.1	-0.19	-0.01
$\mathbf{w_z}[trial]$	0	-0.01	0
α	0.11	0.09	0.14
τ	0.2	0.15	0.26
γ	0.41	0.31	0.51

b

Parameter	P_{50}	P_5	P_{95}
β_S	1.09	0.96	1.22
$\mathbf{w_z}[base]$	-0.1	-0.12	-0.07
$\mathbf{w_z}[block]$	-0.02	-0.08	0.03
$\mathbf{w_z}[type]$	0.17	0.12	0.23
$\mathbf{w_z}[trial]$	0	0	0
$\mathbf{w_z}[prev]$	0.14	0.12	0.16
α	0.12	0.1	0.15
τ	0.44	0.36	0.52
η	0.23	0.18	0.29

c

Parameter	P_{50}	P_5	P_{95}
β_B	0.1	-0.04	0.29
β_V	0.32	0.13	0.48
β_E	0.01	-0.01	0.04
β_S	0.04	-0.05	0.12
κ	0.01	-0.03	0.04
$\mathbf{w_x}[Q_{ch}]$	0.49	-0.5	1.5
$\mathbf{w_x}[V_{trial}]$	-0.76	-1.91	0.38
$\mathbf{w_p}[b]$	0.52	0.51	0.54
$\mathbf{w_p}[Q_{ch}]$	0.69	-0.15	1.54
$\mathbf{w_p}[V_{trial}]$	-0.37	-1.31	0.59
$\mathbf{w_z}[base]$	0	-0.01	0
$\mathbf{w_z}[prev]$	0.12	0.11	0.13
α	0.38	0.23	0.58
ϕ	0.13	0.07	0.22
τ	0.74	0.64	0.84
η	0.57	0.52	0.61
λ	0.53	0.41	0.67

Additional parameter estimates, related to STAR Methods. These tables show estimated population means of model parameters from each study. The median P_{50}, 5th percentile P_5, and 95th percentile P_{95} of the posterior distributions are shown. These parameter estimates are drawn from the primary model for each study; that is, from the model reported in the main article that includes an effect of affect associations A on choice, where A is estimated via Equation 7 (in Studies 1 and 2) or Equation 10 (in Study 3). We only report parameter estimates not already reported elsewhere. In general, β values represent standardized effects of cue associations (e.g., reward associations, affect associations) on choice, **w** values represent unstandardized effects of RL and nuisance variables on valence, and other variables represent learning and decay rates. When reporting the effects of nuisance variables on valence $\mathbf{w_z}$, we use $block$ to refer to the block number, $trial$ to the trial number, $prev$ to the previous valence rating, $type$ to the rating type (post-choice or post-outcome), and $base$ to baseline valence (i.e., the intercept). For more precise definitions of parameters, refer to the equations in which these parameters are introduced; we note these equations in the following. **a.** Parameter estimates from Study 1. β_S is introduced in Equation 8, $\mathbf{w_z}$ in Equation 5, α in Equation 1, τ in Equation S1, and γ in Equation 5. **b.** Parameter estimates from Study 2. β_S is introduced in Equation 8, $\mathbf{w_z}$ in Equation 16, α in Equation 1, τ in Equation S1, and η in Equation S4. **c.** Parameter estimates from Study 3. β values and κ are introduced in Equation 25, **w** values in Equations 9 and 20, α in Equation 1, τ in Equation S1, ϕ in Equation S13, and η in Equation S4. The decay rate λ is described under "Reinforcement learning model selection", above.

Table S7

a

Parameter	Population mean		Population SD	
	M	*SD*	*M*	*SD*
$\beta_{Q,raw}$	1	5	0	5
$\beta_{A,raw}$	0	4	0	6
$\beta_{S,raw}$	2	10	0	10
$\mathbf{w_z}[base]$	0	3	0	5
$\mathbf{w_z}[type]$	0	3	0	5
$\mathbf{w_z}[block]$	0	1	0	1
$\mathbf{w_z}[trial]$	0	1	0	1
α*	-0.05	1.7	0	4
τ*	-0.05	1.7	0	4
γ *	-0.05	1.7	0	4

b

Parameter	Population mean		Population SD	
	M	*SD*	*M*	*SD*
$\beta_{Q,raw}$	1	5	0	5
$\beta_{A,raw}$	0	4	0	6
$\beta_{S,raw}$	2	10	0	10
$\mathbf{w_z}[base]$	0	3	0	5
$\mathbf{w_z}[type]$	0	3	0	8
$\mathbf{w_z}[block]$	0	3	0	8
$\mathbf{w_z}[trial]$	0	0.1	0	0.2
$\mathbf{w_z}[prev]$	0	1.5	0	2
α *	-0.05	1.7	0	4
τ *	-0.05	1.7	0	4
η *	-0.05	1.7	0	4

c

Parameter	Population mean		Population SD	
	M	*SD*	*M*	*SD*
$\beta_{Q,raw}$	0	125	0	175
$\beta_{A,raw}$	0	4	0	6
$\beta_{R,raw}$	0	4	0	6
$\beta_{S,raw}$	0	5	0	10
$\beta_{B,raw}$	0	4	0	6
$\beta_{V,raw}$	0	6	0	8
$\beta_{E,raw}$	0	6	0	8
κ	0	4	0	8
$\mathbf{w_x}[r_{ch}]$	0	40	0	60
$\mathbf{w_x}[b]$	0	0.7	0	1
$\mathbf{w_x}[Q_{ch}]$	0	40	0	60
$\mathbf{w_x}[V_{trial}]$	0	40	0	60
$\mathbf{w_p}[b]$	0	0.7	0	1
$\mathbf{w_p}[Q_{ch}]$	0	40	0	60
$\mathbf{w_p}[V_{trial}]$	0	40	0	60
$\mathbf{w_z}[base]$	0	2	0	4
$\mathbf{w_z}[prev]$	0	1.5	0	2
α *	-0.05	1.7	0	4
ϕ *	-0.05	1.7	0	4
τ *	-0.05	1.7	0	4
η *	-0.05	1.7	0	4
λ *	-0.05	1.7	0	4

Prior distributions for population-level parameters, related to STAR Methods. In all models, we assumed that subject-level parameter values were drawn from normal population-level distributions. The means of the standard deviations of the population-level distributions were estimated from the data. We used normal prior distributions for the population means, and half-normal prior distributions for the population standard deviations. This table shows the mean *M* and standard deviation *SD* of the prior distribution for each population-level parameter. We only report prior distributions used in the primary model for each study; i.e., the model reported in the main article that includes an effect of affect associations A on choice, where A is estimated via Equation 7 (in Studies 1 and 2) or Equation 10 (in Study 3). We constrained parameters marked with an asterisk * to lie between 0 and 1 by transforming them using the logistic function before using them in model calculations. Prior distributions were applied to these parameters before the transformation. β_{raw} values represent unstandardized effects of cue associations (e.g., reward associations, affect associations) on choice. Note that these values differ from β values that lack the "raw" designation, which represent standardized effects (see the section "Affective valence represents outcome values" within the main article for an explanation of how β values were standardized). **w** values represent unstandardized effects of RL and nuisance variables on valence. When reporting the effects of nuisance variables on valence $\mathbf{w_z}$, we use $block$ to refer to the block number, $trial$ to the trial number, $prev$ to the previous valence rating, $type$ to the rating type (post-choice or post-outcome), and $base$ to baseline valence (i.e., the intercept). For more precise definitions of parameters, refer to the equations in which these parameters are introduced; we note these equations in the following. **a.** Priors from Study 1. β_Q is introduced in Equation 1, β_A and β_S in Equation 8, $\mathbf{w_z}$ in Equation 5, α in Equation 1, τ in Equation S1, and γ in Equation 5. **b.** Priors from Study 2. β_Q is introduced in Equation 1, β_A and β_S in Equation 8, $\mathbf{w_z}$ in Equation 16, α in Equation 1, τ in Equation S1, and η in Equation S4. **c.** Priors from Study 3. All β values and κ are introduced in Equation 25. **w** values

are introduced in Equations 9 and 20, α in Equation 1, τ in Equation S1, ϕ in Equation S13, and η in Equation S4. The decay rate λ is described under “Reinforcement learning model selection”, above.

Supplemental References

[S1] Vehtari, A., Gelman, A., and Gabry, J. (2017). Practical Bayesian model evaluation using leave-one-out cross-validation and WAIC. Stat Comput *27*, 1413–1432. https://doi.org/10.1007/s11222-016-9696-4.

[S2] Sinclair, A.H., Wang, Y.C., and Adcock, R.A. (2023). Instructed motivational states bias reinforcement learning and memory formation. Proc Natl Acad Sci U S A *120*, e2304881120. https://doi.org/10.1073/pnas.2304881120.

[S3] Schulz, E., Franklin, N.T., and Gershman, S.J. (2020). Finding structure in multi-armed bandits. Cognitive Psychology *119*, 101261. https://doi.org/10.1016/j.cogpsych.2019.101261.

[S4] Zika, O., Appel, J., Klinge, C., Shkreli, L., Browning, M., Wiech, K., and Reinecke, A. (2024). Reduction of Aversive Learning Rates in Pavlovian Conditioning by Angiotensin II Antagonist Losartan: A Randomized Controlled Trial. Biological Psychiatry *96*, 247–255. https://doi.org/10.1016/j.biopsych.2024.01.020.

[S5] Sivula, T., Magnusson, M., Matamoros, A.A., and Vehtari, A. (2023). Uncertainty in Bayesian Leave-One-Out Cross-Validation Based Model Comparison. Preprint at arXiv, https://doi.org/10.48550/arXiv.2008.10296 https://doi.org/10.48550/arXiv.2008.10296.

[S6] Voodla, A., Uusberg, A., and Desender, K. (2024). Affective valence does not reflect progress prediction errors in perceptual decisions. Cogn Affect Behav Neurosci *24*, 60–71. https://doi.org/10.3758/s13415-023-01147-8.

[S7] Raz, I., Reggev, N., and Gilead, M. (2024). Is it better to be happy or right? Examining the relative role of the pragmatic and epistemic imperatives in momentary affective evaluations. Emotion *24*, 1343–1357. https://doi.org/10.1037/emo0001349.

[S8] Markman, K.D., and McMullen, M.N. (2003). A Reflection and Evaluation Model of Comparative Thinking. https://doi.org/10.1207/S15327957PSPR0703_04.

[S9] Rutledge, R.B., Skandali, N., Dayan, P., and Dolan, R.J. (2014). A computational and neural model of momentary subjective well-being. PNAS *111*, 12252–12257. https://doi.org/10.1073/pnas.1407535111.

[S10] Blain, B., and Rutledge, R.B. (2020). Momentary subjective well-being depends on learning and not reward. eLife *9*, e57977. https://doi.org/10.7554/eLife.57977.

[S11] R Core Team (2024). R: A Language and Environment for Statistical Computing. (R Foundation for Statistical Computing).

[S12] Byrd, R.H., Lu, P., Nocedal, J., and Zhu, C. (1995). A Limited Memory Algorithm for Bound Constrained Optimization. SIAM J. Sci. Comput. *16*, 1190–1208. https://doi.org/10.1137/0916069.
